# Canine Recombinant Adenovirus Vector Induces an Immunogenicity-Related Gene Expression Profile in Skin-Migrated CD11b^+^ -Type DCs

**DOI:** 10.1371/journal.pone.0052513

**Published:** 2012-12-26

**Authors:** Vanessa Contreras, Céline Urien, Luc Jouneau, Mickael Bourge, Coraline Bouet-Cararo, Michel Bonneau, Stephan Zientara, Bernard Klonjkowski, Isabelle Schwartz-Cornil

**Affiliations:** 1 Virologie et Immunologie Moléculaires, Institut National de la Recherche Agronomique, Jouy-en-Josas, France; 2 IMAGIF, Centre National de la Recherche Scientifique, Gif-sur-Yvette, France; 3 UMR Virologie, Institut National de la Recherche Agronomique and Université Paris-Est and Ecole Nationale Vétérinaire d’Alfort and Agence Nationale de Sécurité Sanitaire de l’alimentation de l’environnement et du travail, Maisons-Alfort, France; 4 Centre de Recherche en Imagerie Interventionnelle, Institut National de la Recherche Agronomique, Jouy-en-Josas, France; Commissariat a l’Energie Atomique(cea), France

## Abstract

Gene expression profiling of the blood cell response induced early after vaccination has previously been demonstrated to predict the immunogenicity of vaccines. In this study, we evaluated whether the analysis of the gene expression profile of skin-migrated dendritic cells (DCs) could be informative for the *in vitro* prediction of immunogenicity of vaccine, using canine adenovirus serotype 2 (CAV2) as vaccine vector. CAV2 has been shown to induce immunity to transgenes in several species including sheep and is an interesting alternative to human adenovirus-based vectors, based on the safety records of the parental strain in dogs and the lack of pre-existing immunity in non-host species. Skin-migrated DCs were collected from pseudo-afferent lymph in sheep. Both the CD11b^+^ -type and CD103^+^ -type skin-migrated DCs were transduced by CAV2. An analysis of the global gene response to CAV2 in the two skin DC subsets showed that the gene response in CD11b^+^ -type DCs was far higher and broader than in the CD103^+^ -type DCs. A newly released integrative analytic tool from Ingenuity systems revealed that the CAV2-modulated genes in the CD11b^+^ -type DCs clustered in several activated immunogenicity-related functions, such as immune response, immune cell trafficking and inflammation. Thus gene profiling in skin-migrated DC *in vitro* indicates that the CD11b^+^ DC type is more responsive to CAV2 than the CD103^+^ DC type, and provides valuable information to help in evaluating and possibly improving viral vector vaccine effectiveness.

## Introduction

Gene expression profiling of the *in vivo* innate immune response to vaccines in peripheral blood mononuclear cells (PBMCs) has recently been demonstrated to be predictive of the subsequent magnitude of the antibody and CD8^+^ T cell responses in humans [1], [2]. We reasoned that because dendritic cells (DCs) are key actor cells in bridging the innate and adaptive immune systems, their early gene response might offer an *in vitro* evaluation tool of vaccine vector immunogenicity. In support of this proposal, a recent study showed that the gene expression profile of bone marrow-derived sheep DCs in response to recombinant myxoma virus vectors included many genes that were previously shown to predict vaccine efficacy [3]. As skin DCs and skin-migrated DCs are main targets of nanoparticle-based vaccines that are delivered via the skin route [4], [5], they represent particularly pertinent targets for analyzing the *in vitro* DC response to vaccines. Skin-migrated DCs can be obtained from human or animal skin explants [6] and from afferent lymph in species that are amenable to lymph duct catheterization (ruminants, pigs) [7], [8]. In sheep, skin-migrated DCs collected from pseudo-afferent lymph include two major subsets, the CD26^+^ SIRP^−^ and CD26^−^ SIRP^+^ DCs that were shown to be homologous to the mouse CD8^+^α/CD103^+^ DC and CD11b^+^ DC types respectively, based on transcriptomic and functional assays [9]. The CD26^+^ SIRP^−^ DC subset is specialized in CD8^+^ T cell activation in mixed leukocyte reactions and in soluble antigen cross-presentation [9].

Canine adenovirus serotype 2 (CAV2) is one of the most attractive non-human adenovirus for use as a recombinant vaccine vector [10], [11]. CAV2 represents an alternative to the largely documented human adenovirus serotype 5 (Ad5) vector whose clinical application is impaired due to the frequent pre-existing immunity that interferes with the development of the desired immune response [12], [13]; furthermore, in the context of regulatory constraints in many countries, non-human adenoviruses are likely to be preferred over Ad5 as vaccine platforms for veterinary species. Wild type CAV2 has been used since over 30 years to vaccinate dogs against the Rubarth Hepatitis and has proven to be safe in dogs. A non replicative CAV2 (Cav R^0^) has been generated by deletion of the E1 gene and has been documented to efficiently transduce neurons and to be poorly inflammatory in mice [14]. Several studies document its effectiveness as a recombinant vaccine [15]. For instance, recombinant CAV2 encoding for the Gn protein of the Seoul virus provided long-term immunity against this Hantavirus in mice [16]. Furthermore recombinant CAV2 encoding for the rabies G protein induced protective immunity to rabies in dogs, cats, pigs and sheep with high levels of neutralizing antibodies and T cell responses [17], [18], [19], [20]. The demonstrated immunogenicity of CAV2 and the availability of skin-migrated DCs in sheep led us to assess the gene response profiling of DCs to vaccine vectors in this model system. We found that CAV2 transduced both the CD11b^+^ and the CD103^+^ -type DCs *in vitro,* with a slightly higher efficiency in the later, but only the CD11b^+^-type DC subset presented a broad pattern of gene responses to CAV2 that is associated to dendritic cell maturation, communication between innate and adaptive immune cells, immune cell trafficking, and immune response. Thus our data suggest that the skin-migrated gene response could be used as an *in vitro* tool to help in vaccine design and evaluation of effectiveness.

## Materials and Methods

### Recombinant CAV2 Vectors

The replication-deficient CAV2 vectors used in this study were derived from the Manhattan strain that was deleted of the E1 region. The E1-deleted base vector was named Cav-null R^0^. A E1-deleted vector expressing the Green Fluorescent Protein (GFP) was generated (Cav-GFP R^0^). The VP7 and NS1 sequences from Bluetongue Virus (BTV) Corsican Strain BTV2 were cloned in E1-deleted vector (Cav-VP7 R^0^ and Cav-NS1 R^0^). Cav-GFP, -NS1, -VP7 and Cav-null R^0^ were propagated in DK-E1 cells, were purified by double banding on CsCl gradients and titrated by end-point dilution. Infectious titers were expressed as TCID_50_ ml^−1^.

### Sheep and Ethics Statement

Prealpe female sheep (between 1 and 4 years old) were bred in the Institut National de la Recherche Agronomique (INRA) animal experimentation unit in Jouy-en-Josas, France. Pseudo-afferent prescapular skin draining lymph duct cannulations were performed in sheep as previously described [7]. Low-molecular heparin (enoxaparin (Lovenox), Sanofi-Aventis) was injected intradermally in the shoulder skin every 12 h (2,000 IU anti-Xa per injection). Sheep #61 was vaccinated with commercial BTV2 vaccine (Btvpur Alsap2, Merial), 7 and 3 weeks before CD8^+^ T cells isolation from lymph. The cannulated sheep were housed in the INRA animal facilities in Jouy-en-Josas under the authority of a license issued by the Direction des Services Vétérinaires of Versailles (accreditation numbers 78–93, 78–15, and A78–730). The animal experiment protocols were carried out in strict accordance with the recommendations of the « charte nationale portant sur l’éthique en expérimentation animale » established by the Comité national de réflexion éthique sur l’expérimentation animale (CNREEA, Ministère de l’Enseignement Supérieur et de la Recherche et Ministère de l’Agriculture et de la Pêche). They were approved by the Committees on the Ethics of Animal Experiments of the INRA research center in Jouy-en-Josas and AgroParisTech (approval #11019).

### Lymph Cells Collection

Lymph was collected twice a day in flasks containing 500 IU heparin + 10000 IU penicillin + 10 mg streptomycin. Total lymph cells were spun down at 700g. Low density lymph (LDL) cells were obtained after centrifugation on a 1.065 density iodixanol gradient (Optiprep, Nycomed Pharma, Denmark) as previously described [21].

### Immunolabeling and Flow Cytometry Analyses

Lymph cells were labeled as previously reported [22] using anti ruminant determinant-reacting mAbs, including anti CD1b (Th97A, [IgG2a]), anti CD26 (CC69, [IgG1]), anti CD80 (ILA159, [IgG1]), anti CD86 (ILA190, [IgG1]), anti MHC class 2 (CAT82A, [IgG1]), anti CD40 (ILA156, [IgG1]) and appropriate isotype control (ISC) mAbs. In preliminary experiments of DC activation with Cav-null R^0^ (Figure S1), DCs were isolated from LDL cell preparations by negative selection using Dynabeads as previously described by us [9]. For experiments with DC subsets, the CD26^+^CD1b^+^ (CD103^+^ -type DCs) and CD26^−^CD1b^+^ (CD11b^+^ -type DCs) lymph DCs were sorted from LDL frozen cells by flow cytometry at a 99 % purity level (MoFlo XDP Beckman Coulter). A minimum of 80×10^6^ LDL cells from 6 to 8 successive lymph collections of the same animal were needed to obtain 1.5×10^6^ CD26^+^CD1b^+^ and CD26^−^CD1b^+^ after sorting). In the rest of the study, the CD26^+^CD1b^+^ will be designated as CD103^+^ -type DCs and the CD26^−^CD1b^+^ DCs will be designated as CD11b^+^ -type DCs. Each subset was cultured for 16 hours in mock conditions (7.5×10^5^ cells) or with Cav-null R^0^ (7.5×10^5^ cells, 50 TCID_50_/cell, see below).

### Transduction and Activation of Lymph Cells with Recombinant CAV2 Vectors

Lymph cells (LDL cells or selected DC subsets) were spun down at 700 g at 20°C for one hour with 50 TCID_50_ CAV2 vector per cell to optimize contact of the adenovirus with the target cells [23]. A pilot experiment indicated that 50 TCID_50_/cell was optimal to transduce lymph cells, as used in other comparable studies with adenoviruses [24], [25]. Less than 15 % of the analyzed cells were dead after 36 hours culture and culture with CAV2 had no effect on lymph cell viability. Cells were then cultured for the mentioned time periods in complete medium at 37°C.

### RNA Isolation and Hybridization

Total mRNA from LDL cells (called “reference”) and flow cytometry-purified CD103^+^ -type and CD11b^+^ -type DCs mock -cultured or cultured with Cav-null R^0^ for 16 hours were extracted using the Qiagen RNeasy Micro Kit and checked for quality on an Agilent 2100 Bioanalyzer with RNA 6000 Nano Chip kit (Agilent). Four independent sheep were used for the microarray data generation, corresponding to a total of 16 RNA samples (mock CD103^+^ -type DCs, Cav-null R^0^ + CD103^+^ -type DCs, mock CD11b^+^ -type DCs, Cav-null R^0^ + CD11b^+^ -type DCs). Total RNAs (200 ng) were amplified by linear PCR and the amplification products were labeled with Cy3 using Bioprime Array CGH Genomic Labelling System Kit (Invitrogen, Carlsbad, CA). RNA from the LDL reference preparation was similarly amplified, labeled by Cy5 and used as a reference probe for the hybridization. Each Cy3 labeled cDNA was co-hybridized with the Cy5 reference probe onto ovine 15K 60-mers microarrays (15208 probes, Reference: G4813A, AMADID: 19921, Agilent) following the manufacturer’s protocol. Raw data were extracted from scanned microarray images (.tif) using Feature Extraction Software v9.5 (Agilent) and normalized using the Quantile method adapted to bicolour microarrays. All the protocols used can be obtained by contacting the microarray and sequencing platform of the IGBMC (see also the web site http://www-microarrays.u-strasbg.fr).The microarray data have been assigned the Gene Expression Omnibus number GSE42502 (www.ncbi.nlm.nih.gov/geo/info/), and they are publicly available.

### Microarray Analysis

The probe signals corresponding to genes significantly induced by Cav-null R^0^ over mock conditions was analyzed by a linear model [26] taking into account treatment of the samples (Cav-null R^0^ versus mock) and the origin (animal) of the samples using a LIMMA analysis. The p-values obtained have been corrected for multiple testing using the Benjamini and Hochberg procedure [27]. By convention, genes were considered dys-regulated by the Cav-null R^0^ treatment when their corresponding Agilent probe signal showed a > 2 or < 0.5 fold modulation compared to the mock condition, with an adjusted p-value < 0.05. The ovine microarray has been partially annotated by the SIGENAE group (INRA - SIGENAE [http://www.sigenae.org/]). Regarding the differentially expressed genes revealed by non annotated probes, the corresponding EST were blasted against bovine sequences (RefSeq Bos Taurus) in order to identify the putative bovine orthologous gene (>200 nt with >92 % identity). The visual representation of the different experimental condition effects (Cav-null R^0^ treatment, cell subsets, animal) was given by the unsupervised Principal Component Analysis, using the FactoMineR package.

For functional analysis, the gene list selected by the filter was completed by genes checked by qRT-PCR that also showed LIMMA p-values < 0.05 (Table S1 and S2) and the final CAV2-modulated gene data set was subjected to Ingenuity Pathway Analysis (IPA, Ingenuity Systems, www.ingenuity.com). The molecules from the CAV2-modulated gene data set were associated with Biological Functions in the Ingenuity Knowledge Base. Right‐tailed Fisher’s exact test was used to calculate a p‐value determining the probability that each biological function assigned to the data set is due to chance alone. In addition, the recently released version of IPA offers functional annotations of downstream effects of gene expression data. The downstream effect analysis of IPA examines genes in the dataset that are known to affect biological function and compares their direction of change to what is expected from the literature. When the direction of change is consistent with the literature across the majority of genes, then the function is predicted to be increased in the biological sample (z-score > 2), whereas if the direction of change is mostly anti‐correlated with the literature then the function is predicted to be decreased in the biological sample (z-score < -2).

The CAV2-modulated gene data set was also subjected to the Ingenuity Canonical Pathway Analysis that identifies the pathways from the IPA Knowledge Base library of canonical pathways that are most significant to the data set. The significance of the association between the data set and the canonical pathway was measured in 2 ways: 1) A ratio of the number of molecules from the data set that map to the pathway divided by the total number of molecules that map to the canonical pathway is displayed. 2) Fisher’s exact test was used to calculate a p‐value determining the probability that the association between the genes in the dataset and the canonical pathway is explained by chance alone.

Finally, the CAV-2 modulated gene data set was overlaid onto a global molecular network developed from information contained in the Ingenuity Knowledge Base. Networks were algorythmically generated based on their connectivity. Molecules are represented as nodes and the biological relationship between two nodes is represented as a line. All lines are supported by at least one reference from the literature from a textbook, or from canonical information stored in the Ingenuity Knowledge Base. The intensity of the node color indicates the degree of up‐ (red) regulation. Nodes are displayed using various shapes that represent the functional class of the gene product.

### RT-PCR

RNA (400 ng) was reverse transcribed using random primers and the Multiscribe reverse transcriptase (Applied Biosystem). Quantitative real-time PCR was carried out using 100 ng cDNA with 300 nM primers in a final reaction volume of 25 µl of 1×SYBR Green PCR Master Mix (Applied Biosystem). The primers used to amplify the ovine IL12 (Forward GAATTCTCGGCAGGTGGAAG, reverse GTGCTCCACGTGTCAGGGTA), IL6 (Forward GCTGCTCCTGGTGATGACTTC, Reverse GGTGGTGTCATTTTTGAAATCTTCT), TNFα (Forward CAAGGGCCAGGGTTCTTACC, reverse GCCCACCCATGTCAAGTTCT), IL2 (Forward GTGAAGTCATTGCTGCTGGA, reverse TGTTCAGGTTTTTGCTTGGA), IL18 (Forward GAGCACAGGCATAAAGATGG, reverse TGAACAGTCAGAATCAGG CATA), MX1 (Forward TGCGCATGGCTCAGGAT, reverse CCAGATCGGGCTTTGTCAAG), ETS2 (Forward TGCAGCAAGGCTGTGATGAG, reverse CGGCGTTGCTCCTTTTTG), TNFRSF13 (Forward GGACGCTAAGATATCGCGAGAT, reverse TGTGCGCAGGTCACAGAAG), EIF2AK2 (Forward AGGTTGGTCAAGGATTTCACAGA, reverse TCTGTGTTCGGCTTGAAAACTC), STAT1 (Forward ACCCCGGAATCTGTCCTTCT, reverse CAGCTCAGCACCTCTGAAAGC), Caspase1 (Forward GCCAAATCTGCA TCAGCCATA, Reverse CTGAAGTGAGCCCCAGTATTCC), IRF1 (Forward AAGGACGCCTGTCTGTTT CG, reverse TCCTTTTCCCCTGCTTTGTATC), IL-8 (Forward TTCCAAGCTGGCTGTTGCTCTCTT, reverse GCATTGGCATCGAAG TTCTGTACTC), CCL5 (Forward GTGGGTGCGAGAGTACATCAAC, reverse GGCGCAAGTTCAGGTTCAAG), CXCL10 (Forward AGAGCCTTCAAAGCCTGTTTCTT, reverse TCCTTTTCATTGTGGCAATAATCTC), IRF7 (Forward GGCAAGTGCAAAGTCTACTGG, reverse GAAGTCAAAGATGGGCGTGT), CCR7 (Forward CCAGATGGTGGTAGGCTTCCT, reverse GCGGATGATGACAAGGTAGCA) were designed using the primer express software (v2.0). PCR cycling conditions were 95°C for 10 min, linked to 40 cycles of 95°C for 15 sec and 60°C for 1 min. Real-time PCR data were collected by the ABI 7900HT Sequence Detection System (Applied Biosystem) and 2-*Δ*CT calculations for the relative expression of the different genes (arbitrary units) were performed with the SDS 2.1 software (Applied Biosystem) using GAPDH for normalization.

### Antigen Presentation to Immune Autologous CD8^+^ T cells

CD103^+^ and CD11b^+^ -type DCs were sorted as described above from sheep #61 afferent skin lymph. CD8^+^ T cells, that recirculate in skin-lymph, were selected from afferent lymph on the same sheep, using the CAT80C and 7C2 anti sheep CD8 mAbs and goat anti mouse IgG Miltenyi magnetic beads, 3 weeks after the recall injection of commercial BTV8 vaccine. The CD103^+^ and CD11b^+^ -type DCs (2×10^4^) were pulsed overnight with 50 PFU/cell Cav-NS1 R^0^ and Cav-VP7 R^0^ oρ ωith Cav-null R^0^ (control) or left alone. Autologous BTV-immune CD8^+^ T cells (2×10^5^) were added to the wells and the co-culture was pursued for 72 hours. Supernatants were harvested for ruminant IFN**γ** detection by ELISA as described [9].

### Statistics

Statistical significance of transduction and functional tests was estimated with paired Student’s t test. The statistics of the microarray analyses are described in the corresponding section.

## Results

### The CAV2 Vector Transduces both CD103^+^ and CD11b^+^ -type DCs

**Figure 1:**
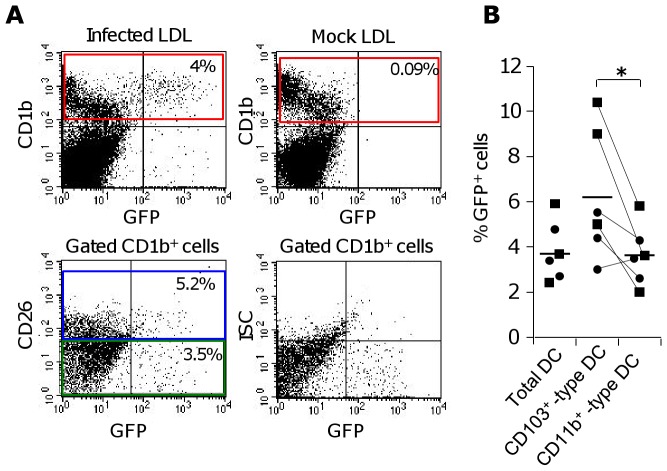
CD103^+^ -type and CD11b^+^ -type DCs are transduced by Cav-GFP R^0^. (A) LDL cells were cultured alone or infected for 36 hours with Cav-GFP R^0^ (50 TCID_50_/ml). The percent of GFP^+^ cells were detected within the CD1b^+^ (red rectangle), the CD26^+^ (CD103^+^ -type, blue rectangle) and the CD26^−^ (CD11b^+^-type, green rectangle) DCs. Representative FACS profiles are shown, including the isotype control (ISC) corresponding to the CD26 staining. (B) Six independent experiments described in (A) were performed with 2 different sheep (▪ #64 and •#80). Statistical significance (paired Student t-test) is indicated by * with p < 0.05.

We evaluated whether CAV2 can transduce the skin-migrated sheep DC subsets collected from afferent lymph. A pilot experiment showed that 50 TCID_50_ of Cav-GFP R^0^/cell was optimal to induce transduction of low density lymph (LDL) cells that are enriched in DCs (data not shown). As shown Fig. 1A in LDL cells, the GFP transgene encoded by CAV2 was found mainly expressed in CD1b^+^ cells that correspond to DCs in sheep afferent lymph [7] and CD1b^−^ cells appeared little transduced (Fig. 1A). From 6 independent infections using LDL cell preparations from 2 sheep, we found that 3.8 ± 0.8 % of the skin-migrated DCs were transduced by Cav-GFP R^0^ (Fig. 1B). Cav-GFP R^0^ transduced a slightly higher percentage of cells in the CD103^+^ -type DCs subset than in the CD11b^+^ -type DCs that represent the major part of the skin-migrated DCs (> 85%) [7] (Fig. 1B, 6.2 ± 2.3 versus 3.6 ± 0.9, p<0.05). Despite a higher receptivity of CD103^+^ -type DCs to CAV2, both FACS-sorted subsets when transduced with Cav R^0^ expressing Bluetongue NS1 and VP7 antigens, were capable of activating autologous immune CD8^+^ T cells, with the CD11b^+^ -type DCs being slightly more efficient (Fig. S1). Thus a small but significant fraction of skin-migrated DCs are transduced by CAV2 vector and can directly stimulate autologous CD8^+^ T cells.

### The CAV2 Vector Triggers a Broad Gene Response in Skin-migrated DCs, Especially in the CD11b^+^ -type DC Subset

We next analyzed the global gene response of CD11b^+^ and CD103^+^ -type DCs to CAV2 in order to evaluate whether this gene response could be associated to the immunogenicity of the vector. In these experiments, an « empty » CAV2 vector, Cav-null R^0^, was used to conduct the analysis independently of any transgene expression. Preliminary experiments were done to determine the optimal time point for analyzing the DC response to Cav-null R^0^ *in vitro* (Fig. S2A). For all the tested cytokine genes (*TNFA, IL6, IL18, IL12* and *IL2*), the optimal time point to detect the gene upregulation by Cav-null R^0^ was 16 h. We also found that the overall population of DCs expressed higher co-stimulatory CD80 and CD86 molecules on the surface upon Cav-null R^0^ stimulation, indicating that skin-migrated DCs can be further maturated during the *in vitro* culture with vector and that DC activation concerns a large DC fraction, beyond the transduced ones (Fig. S2B and S2C).

**Figure 2:**
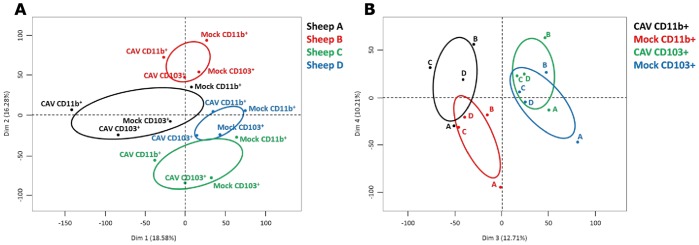
Principal component analysis of microarray data from the CD103^+^ and CD11b^+^-type DCs activated by the CAV2 vector. The expression value for each gene in mock and CAV2-activated CD103^+^ and CD11b^+^-type DCs from 4 sheep (A: sheep #58; B: sheep #74, C: sheep #66, D: sheep #61) were used for the analysis. The expression values for the 4 first components (Dim 1, 2, 3, 4) of the principal component analysis were plotted. (A) The Dim 1 and dim 2 axes may be interpreted as the “animal” component representing 18.58 % and 16.28 % of the microarray data variance; CD11b^+^ and CD103^+^ stands for CD11b^+^ and CD103^+^ -type DCs, Mock stands for DCs cultured alone, CAV stands for Cav-null R0 treated DCs. (B) the Dim 3 may be interpreted as the “subset” component (12.71 % of the array data variance) and dim 4 represents the “CAV2 treatment” component (10.21 % of the array data variance).

**Figure 3:**
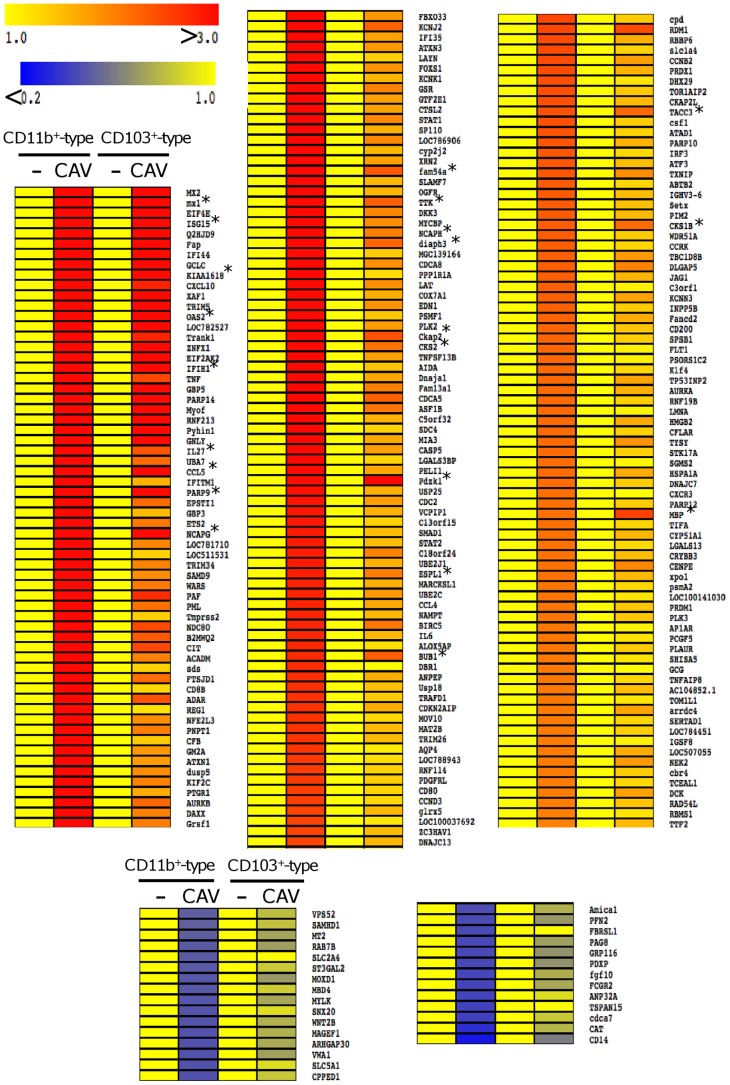
Transcriptional response to CAV2 vector in the CD103^+^ and CD11b^+^ -type DC subsets. (A)The mean fold induction of the selected genes up-regulated by Cav-null R^0^ are represented in graded yellow to red color (from 1 to >3) for both CD103^+^ and CD11b^+^ -type DCs from 4 sheep. The represented genes were selected from the significantly induced genes in the CD11b^+^ -type DC subset (222 genes, p<0.05, fold > 2) and they were ranked based on their fold induction in the CD11b^+^ -type DC subset. The significantly activated genes in the CD103^+^ subset (21 genes, p<0.05, fold > 2) are indicated by a star. (B) The mean fold reduction levels of the selected down-modulated genes by Cav-null R^0^ are represented in graded yellow to blue color (from 1 to <0.2) for both CD103^+^ and CD11b^+^ -type DCs from 4 sheep. The represented genes were selected from the significantly reduced genes in the CD11b^+^ -type DC subset (29 genes, p<0.05) and they were ranked based on their fold induction in the CD11b^+^ -type DC subset. No gene expression was significantly reduced in the CD103^+^ -type DC subset.

The comparative transcriptomic response of flow cytometry-sorted CD11b^+^ and CD103^+^ -type DCs to Cav-null R^0^ (50 TCID_50_/cell) was established from 4 different sheep after 16 h culture. We used the ovine Agilent microarrays that include 15000 ovine long 60-mers. They are the only commercially available gene arrays for sheep that partially cover the sheep genome that is not yet annotated [28]. The gene expression analysis of the CD11b^+^ and CD103^+^ -type DCs response to Cav-null R^0^ was informative. In order to visualize the different experimental conditions effects, the normalized probe signals of each mock and CAV2-activated DC subset were subjected to principal component analysis. As shown Fig. 2A, a strong “animal effect” is observed, that is expected from studies with primary cells from outbred animals. Despite this strong animal effect, Fig. 2B shows that the samples grouped according to the CAV2 treatment (Y-axis, dim 4) and to the DC subset type (x-axis, dim 3). The lists of the genes dys-regulated by Cav-null R^0^ were established for the CD11b^+^ and CD103^+^ -type DCs using the LIMMA R package and the p-values have been adjusted for multiple testing using the Benjamini-Hochberg procedure. The up- and down-modulated genes were selected when they presented a > 2-fold and < 0.5-fold modulated expression. In the CD11b^+^ -type DCs, 222 up-regulated genes (Table S1) and 29 down-modulated genes were identified (Table S2). In the CD103^+^ -type DCs, only 21 up-regulated genes were found (Table S1). The heat map representation of the expression levels of the 222 up-regulated genes and 29 down-modulated genes (Fig. 3) shows that all the up-regulated genes in the CD103^+^ -type DCs are also up-regulated in the CD11b^+^ -type DCs, and usually at a higher extent in the latter. Thus overall, our result show that CD11b^+^ -type DCs are more transcriptionnaly activated by Cav-null R^0^ than are the CD103^+^ -type DCs, and that no gene is specifically modulated in the CD103^+^ -type DCs.

**Figure 4:**
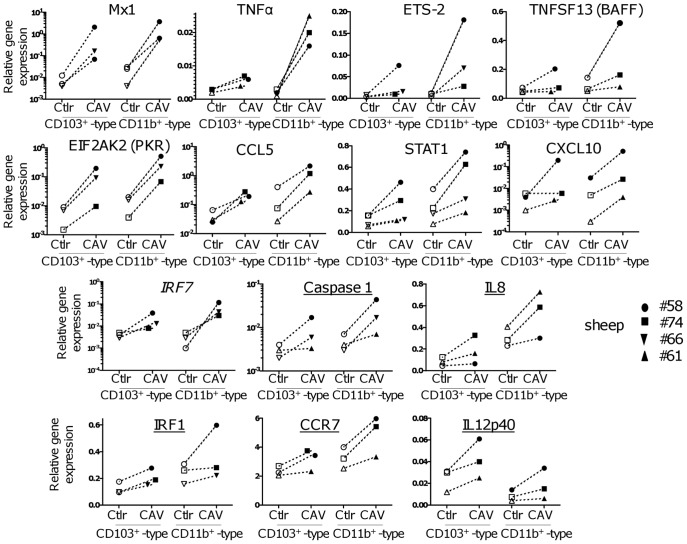
Validation of gene expression induced by CAV2 vector with qRT-PCR. A selection of genes was checked for CAV2-induced upregulation using qRT-PCR on cDNA from the CD103^+^ and CD11b^+^ -type DCs (4 different sheep were used in total, 3 per validated gene). Each gene detection was normalized with GAPDH expression and the relative gene expressions (log2(CTgene-CTGAPDH)) are reported. The gene names printed with regular font were selected as being up-regulated in the CD11b^+^ type DC microarrays (p < 0.05 ANOVA followed by Benjamini-Hochberg FDR and > 2 fold), the gene names printed in italic are not represented by any probe on the ovine microarray, the underlined gene names were close to be selected by the variance analysis in the CD11b^+^ -type DCs.

The transcriptomic results provided by the LIMMA analysis were confirmed and completed by qRT-PCR analysis using 3 to 4 different sheep. As shown in Table S1 and Fig. 4 for the CD11b^+^ -type DCs, *MX1, TNFΑ, ETS2, TNFSF13B, CCL5, STAT1, CXCL10, EIF2AK2 (PKR)* gene expressions were found up-regulated by the LIMMA selection and were confirmed by qRT-PCR (Fig. 4). For the CD103^+^ -type DCs, only MX1 of this qRT-PCR analysis was selected by the LIMMA filter. However, the other gene mRNAs were also up-regulated but at a clearly lower extent than in CD11b^+^ -type DCs, and often between 1.5 and 2 folds (Table S1).

As our goal was to evaluate the immunity-related functions induced in DCs by Cav-null R^0^, we extended our qRT-PCR analysis to genes that were close to be selected by the LIMMA and the 2-fold up-regulation filters and that could possibly play a role in CAV2 vector immunogenicity (bottom part in italic of Table S1 and Fig. 4). We found that in most sheep cases, *CASP1, IL8, IRF1, CCR7* and *IL12B* levels were indeed up-regulated by Cav-null R^0^. The *IL12B* gene was expressed at a higher basal level in the CD103^+^ -type DCs and *IL8* was found expressed at higher basal levels in the CD11b^+^ -type DCs in comparison to the expression in the counterpart subset, as previously published [9]. However *IL12B* and *IL8* were found similarly activated by Cav-null R^0^ in both subsets (Fig. 4). Finally, as several genes of the IFN pathways appeared up-regulated by Cav-null R^0^, we tested *IRF7* gene activation by qRT-PCR analysis, because no probe for *IRF7* were present on the ovine gene chip. *IRF7* was found up-regulated by Cav-null R^0^ in both CD103^+^ (not significant with Student t-test) and CD11b^+^ -type DCs (Fig. 4, p < 0.05 using Student t-test).

### The *in vitro* Gene Responses of Skin-migrated DC Subsets to the CAV2 Vector is Associated to Immune Response Activation

The CAV2-modulated genes in CD103^+^ and CD11b^+^ -type DCs completed with the genes confirmed by qRT-CR (i.e. first list + *IL8, IRF1, CCR7, IL12B, IRF7*) were submitted to the Ingenuity Pathway Analysis (IPA, http://www.ingenuity.com/) to determine the statistical over-representation of functional gene clusters and the functional annotations of downstream effects of the gene expression data (see material and methods). The analysis of dys-regulated gene list in Cav-null R^0^ stimulated CD11b^+^ -type DCs provided z-scores that predicted the activation of several functions based on the knowledge data base of IPA (Table 1). As found in previous transcriptomic analyses with human adenovirus [29], the function “Proliferation of cells” is predicted to be activated in the CD11b^+^ -type DCs. Many molecules in the “Proliferation of cells” list are also found in the “Cell death” function list, although this latter function is not activated (z-score = 1.67). Four functional clusters related to immunity are predicted to be activated in the CD11b^+^ -type DCs, and correspond to “activation of immune responses” (z-score = 2.4), “inflammation” (2.16) “cell movement of myeloid cells” (2.06) and “activation of mononuclear leukocytes” (2.07). Conversely none of these functions were predicted to be activated in the CD103^+^ -type DCs, despite the fact that several immune genes are stimulated in this subset such as *CCL5, IFIH1, IL27, ISG15*, and *MX1*. In this subset, only the “cell proliferation and cell death” functions were significantly predicted to be activated (z-score 2.34 and 1.98).

**Table 1:** Biological functions that are predicted to be activated by CAV2 vector in CD103^+^ and CD11b^+^ -type DCs.

Function annotation	CD11b^+^ -type DCs				CD103^+^ -type DCs			
	Molecules	Z-score	P-value	#	Molecules	Z-score	P-value	#
Proliferation of cells	ANP32A,ANPEP,ATF3,AURKA,AURKB,BIRC5,BUB1 CASP1,CAT,CCL4,CCL5,CCND3,CCR7,CD14,CD80 CDCA7,CDK1,CFB,CFLAR,CKS1B,CKS2,CSF1 ,CTSL2,CXCL10,CXCR3,CYP2J2,DAXX,DCK,EDN1,EIF2AK2,EIF4E,ETS2,FGF10,FLT1,GCG,GCLC,HMGB2,HSPA1A/HSPA1B,IFITM1,IL12B,IL27,IL6,IL8,IRF1,ISG15,JAG1,KCNJ2,KIF2C,KLF4,LAT, MARCKSL1,MBP,MT2A,MYCBP,MYLK,NAMPT,NCAPG,NEK2, PARP10,PDZK1,PELI1,PIM2,PLAUR,PLK3,PML,PRDM1,PRDX1, SDC4,SERTAD1,SLAMF7,SMAD1,STAT1,TACC3,TNF,TNFAIP8, TNFSF13B,TOM1L1,TTK,TXNIP,UBE2C,USP18,WARS	2.488	4.71E-11	82	BUB1,CCL5,CKS2,IL27,ISG15,NCAPG,PDZK1,PIM2,TTK	1.97	8.4E-03	9
Immune responses	ALOX5AP,AMICA1 *,ANPEP,ATF3,CASP1,CASP5,CCL4,CCL5, CCR7,CD14,CD200,CD80 ,CFB,CSF1 ,CTSL2,CXCL10,CXCR3,EDN1,EIF2AK2,FLT1,GM2A,GNLY,IFI44, IFIH1,IL12B,IL27,IL6,IL8,IRF1,IRF3,IRF7,ISG15,KLF4,LAT,MBP, MT2A,MX1,MYLK,PELI1,PLAUR,PML,PRDM1,SLC2A4,STAT1, STAT2,TNF,TNFSF13B,TRIM5,TXNIP,ZC3HAV1	2.454	5.33E-07	50				
Inflammation	ALOX5AP,ATF3,CASP1,CASP5,CCL4,CCL5,CCR7,CD14,CD200, CD80,CFB,CTSL2,CXCL10,CXCR3,EIF2AK2,FLT1,IL12B,IL27,IL6, IL8,LAT,MT2A,PLAUR,SLC2A4,STAT1,TNF,TNFSF13B	2.163	3.10E-07	27				
Cell movement of myeloid cells	ALOX5AP,AMICA1,CASP1,CCL4,CCL5,CCR7,CFB,CSF1 CTSL2,CXCL10,CXCR3,EDN1,FLT1,GNLY,IL12B,IL6,IL8,MYLK, PLAUR,TNF	2.081	4.00E-05	20				
Activation of mononuclear leukocytes	CCL4,CCL5,CCR7,CD14,CD80,CSF1 ,IL12B,IL27,IL6,IL8,IRF1 ,LAT,MBP,PELI1,STAT1,TNF,TNFSF13B,TXNIP	2.071	1.26E-04	18				
Cell death	ADAR,ANP32A,ANPEP,ATF3,ATXN1,ATXN3,AURKA,BIRC5,BUB1 ,CASP1,CASP5,CAT,CCL4,CCL5,CCND3,CCR7,CD14,CD200, CD80, CDK1,CDK20,CFLAR,CIT,CKAP2,CSF1 ,CTSL2,CXCL10,CXCR3,CYP2J2,DAXX,DCK,DKK3,DNAJA1,DUSP5,EDN1,EIF2AK2,EIF4E,ESPL1,ETS2,FANCD2,FAP,FGF10,FLT1,GCG,GCLC,GNLY,GSR,HMGB2,HSPA1A/HSPA1B,IFIH1,IL12B, IL27,IL6,IL8,INPP5B,IRF1 IRF3,ISG15,JAG1,KLF4,LAT,LGALS3BP,LMNA,MBD4,MBP,MT2A,MX1,MYLK,NAMPT,NDC80,NEK2,PDZK1,PIM2,PLAUR,PLK2, PLK3,PML,PNPT1,PRDM1,PRDX1,PTGR1,RAD54L,RBBP6, RNF19B,SDC4,SETX,SGMS2,SHISA5,SLAMF7,SLC2A4,SMAD1, SP110,STAT1,STAT2,STK17A,TACC3,TNF,TNFAIP8,TNFSF13B, TTK,TXNIP,UBA7,UBE2C,USP18,XAF1,XPO1	1.67	1.14E-15	98	BUB1,CCL5,CKAP2,ESPL1,IFIH1, IL27,ISG15,MX1,PDZK1,PIM2,TTK	1,742	3.68E-03	11

* the expression of the underlined molecules is down-modulated.

**Figure 5:**
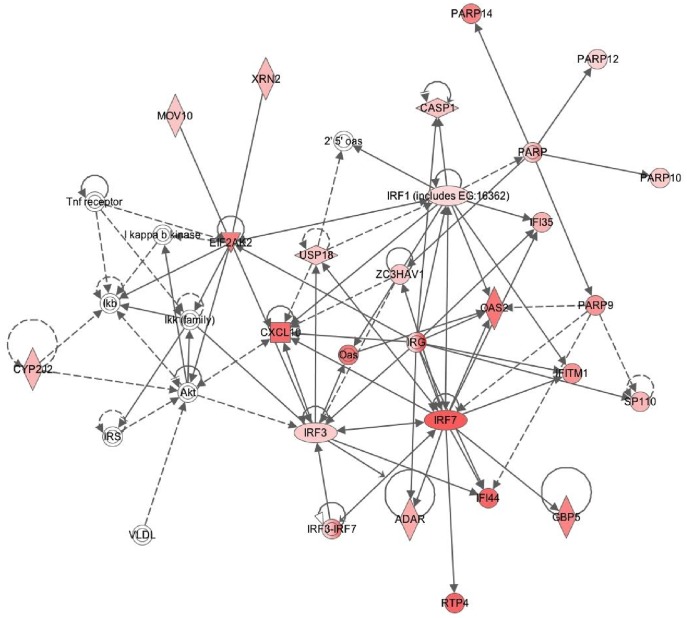
Antimicrobial gene network induced by CAV2 vector in CD11b^+^ -type DCs. An antimicrobial gene network centered on IFN was generated by the Ingenuity Pathways Analysis on the selected genes dys-regulated by CAV2 vector in CD11b^+^ -type DCs (Table S1, p < 0.05 and fold > 2, or p < 0.05 and folds confirmed by qRT-PCR). Molecule types are represented by symbols: diamonds (enzymes), triangles (kinase), square (cytokine), double circle (complex), oval (transcription regulator), circle (others).

Canonical pathways induced by Cav-R^0^ in the CD11b^+^ -type DCs were also revealed by IPA (Table 2). The top ones were the “communication between innate and adaptive immune cells” (p-value = 10^−7.72^) and the “activation of IRF by cytosolic pattern recognition receptors” (p-value = 10^−7.05^) and “interferon signaling” (p-value = 10^−5.4^) (Table 2). None of these pathways were found significantly activated in the CD103^+^ -type DCs. Finally the most meaningful biological network revealed by IPA in CD11b^+^ -type DCs corresponded to the “Antimicrobial, Inflammatory and Infection” network (23 up-regulated genes over the 39 members of the corresponding IPA network) that was centered on IFN related pathways (Fig. 5).

**Table 2:** Top canonical pathways induced by CAV2 vector in CD103^+^ and CD11b^+^ -type DCs.

Canonical pathways	CD11b^+^ -type DCs		CD103^+^ -type DCs	
	Genes	-log(p-value)	Genes	-log(p-value)
Communication between Innate and Adaptive Immune Cells	CXCL10,IL8,CCL4, CD80,IL12B,CCL5, IL6,TNF,CD8B,CCR7,TNFSF13B	7.72E00		
Activation of IRF by Cytosolic Pattern Recognition Receptors	IFIH1,IRF7,STAT2, IRF3,IL6,STAT1,ADAR,TNF,ISG15	7.05E00	IFIH1,ISG15	2.6E00
Interferon Signaling	IFITM1,MX1,IFI35, STAT2,STAT1,IRF1	5.43E00	MX1	1.4E00
Crosstalk between Dendritic Cells and Natural Killer Cells	CD80 (includes EG:12519),MICB, IL12B,IL6,TNF,CCR7	3.02E00		
Dendritic Cell Maturation	CD80 ,IL12B,STAT2,IL6, STAT1,TNF,CCR7	2.42E00		

Altogether, our results show that the *in vitro* gene response of skin-migrated CD11b^+^ -type DCs to Cav-null R^0^ is broad and clusters into activated functions that are related to the immunogenicity.

## Discussion

In this study, the global gene response profile of skin-migrated DCs revealed that CAV2 stimulation *in vitro* induces an immunogenicity-related gene expression profile primarily in the CD11b^+^ -type DCs, whereas both CD103^+^ and CD11b^+^ -type DCs are transduced by the vector.

Skin-migrated DCs were significantly transduced by CAV2 *in vitro*, contrasting with the refractory state of human monocyte derived-DCs (Mo-DCs) to CAV2 transduction [24], when used at similar multiplicity of infection. Mo-DCs and skin-migrated DCs are distinct DC types: the later originate from common DC precursors in the bone marrow [30], whereas the former are derived from monocytes cultured with GM-CSF and IL-4 *in vitro*. Indeed several groups have reported that skin-derived DCs, and especially skin-migrated DCs, are more permissive to Ad5 or Ad35 transduction than Mo-DCs [6], [31]. Furthermore, skin-migrated DCs from cattle were previously found to be highly susceptible to Ad5 transduction [25]. Difference of species (human/sheep) may also explain the discrepancy of the results for CAV2 transduction in sheep skin-migrated and human Mo-DCs, that may implicate differences in viral receptors and/or intracellular trafficking. For instance Ad35 shows species-specific interactions with target cells, due to the selective expression of its receptor CD46 in primate cells [32]. Nevertheless, the percent of sheep skin lymph DCs transduced by CAV2 remained relatively low in the two CD103^+^ and CD11b^+^ -type DCs (6.2 and 3.6 % respectively). Of note, the increase in co-stimulatory molecules expression could be detected in the overall DC population cultured with CAV2, much beyond the limited fraction of transduced cells. Thus it is likely that CAV2 activates DCs via sensing mechanisms that do not require complete transduction by the vector. Indeed UV-inactivated Ad5 activates DCs to the same extent as non UV-irradiated Ad5 for the production of inflammatory cytokines and NF-κB activation [33]. Sensors such as the heparin-sensitive adenovirus receptor [34], TLR9 [13] and the helicase DDX41 [35] that are involved in Ad5 sensing need to be tested for their implication in CAV2 signaling in DCs.

The direct transduction of skin-migrated DCs by CAV2 observed in our *in vitro* experiments may participate in CAV2 immunogenicity but may not be the key mechanism of CAV2 vector immunogenicity *in vivo*. Indeed in the case of Ad5 in mice, DCs were required for induction of immunity to transgene but their direct transduction seemed not to be required for inducing immunity [36]. In addition Hangalapura et al documented that Ad5 essentially transduced non DC cells when given intradermally [37]. The preferential transduction of non DC cells may possibly due to their expression of the Coxsachie and Adenovirus Receptor (CAR), the Ad5 primary receptor, whereas DCs do not express CAR and use a not yet defined fiber shaft receptor [34]. In the case of CAV2 that also utilizes CAR to transduce epithelial and neuronal cells [38], the transduction of non DC cells in the skin may play an important role in its immunogenicity, leading to cross presentation by the DCs in the lymph node [36]. Notably the general activation of the skin-migrated DCs by CAV2, that we report here, may allow the proper priming of DCs for triggering efficient antigen presentation both after their direct transduction and after capture of non DC transduced cell fragments (cross presentation).

The distinct capacities of the two skin-migrated DC subsets to induce gene responses to CAV2 cannot be explained by difference in transduction efficiency because CD103^+^ -type DCs are better transduced by CAV2 than are the CD11b^+^ -type DCs (Fig. 1). We can not exclude that technical artifacts such as differential response/activation to sorting conditions or differential timing for optimal response explain the differences of the DC subset response to CAV. However despite limited global gene activation in response to CAV2, the CD103^+^ -type DCs still presented their intrinsic properties, with higher IL-12p40 mRNA basal levels and further increase upon CAV2 stimulation (Fig. 4). In addition, several genes were induced under our CAV2 activating conditions in the CD103^+^ -type DCs, at the same or higher extent than in the CD11b^+^ -type DCs (*IL12p40, PDZK1, TACC3, CKS1B, MBP, IL8, IRF7, IRF1,* Table S1 ) supporting that the CD103^+^ -type DCs kept its functionality and capacity for being activated. Indeed the CD103^+^ -type DCs were efficient at activating antigen-specific CD8^+^ T cells upon CAV2 stimulation (Fig. S1). In addition, the viability of both DC subsets cultured with CAV2 is similar (>90% as measured by 7-Amino-Actinomycin D exclusion, data not shown) and both subsets show similar z-scores for the cell death function in the 2 subsets (Table 1). The higher and broader gene response to CAV2 stimulation encountered in the CD11b^+^ -type DCs might be explained by the different expression of viral sensors in the two subsets. Indeed murine CD8α^+^ and CD11b^+^ DCs [39] and sheep lymph DC subsets [40] express different sets of TLR receptors. In addition, murine CD8α^+^ DCs largely lack the receptors required to sense certain viruses in the cytoplasm, such as retinoic acid-inducible gene I [41], [42]. However we could not detect consistent differences between the two lymph DC subsets in the mRNA expression of known adenovirus sensors, (our microarray data, not shown). Other yet unknown adenovirus sensors may be involved, and proteomic studies are also needed to detect differences of sensor expression.

Our data suggest that the *in vitro* transcriptomic signature of DCs in response to vaccines may bring information on the possible downstream immunogenicity. This approach brings complementary information to classical functional *in vitro* assays. Indeed, while a direct antigen presentation assay by CAV2-transduced CD103^+^ and CD11b^+^ -type DCs showed a slightly higher efficiency of the later DC type for stimulating antigen-specific CD8^+^ T cells, the transcriptomic analysis provided a broad information on the global DC subset activation by the vector, that may be even more pertinent if indirect antigen presentation is concerned. Indeed the transcriptomic signature unraveled that CAV2-stimulated CD11b^+^ -type DCs for B and T cell activation and immune cell trafficking. The signature that we obtained here would be strengthened and completed by using RNAseq strategy as the limited array available for sheep studies probably bias the results. Finally subsequent studies need to be conducted to establish gene expression signatures in DCs *in vitro* that predict the magnitude of the immune response using formal training and validation test processes. These transcriptomic signatures may vary with vectors and vaccine formulations. DC gene response signatures to different vaccine vectors and formulas may importantly help in vaccine improvement.

Altogether, this work indicates that CAV2 activates the CD11c^+^ -type and not the CD103^+^ -type DCs to express a transcriptomic signature related to stimulation of immune response. Thus the analysis of the DC gene responses *in vitro* stands as an interesting and convenient assay to evaluate and possibly improve vaccine effectiveness. The *in vitro* innate gene response of DCs combined to other parameters such as the strength and duration of transgene expression, structural analyses of predictive antigenicity of the transgene-encoded protein, and classical immunological functional assays could altogether allow prediction of downstream immunogenicity and offer new avenues to reasoned vaccine developments, while reducing the number of animal tests.

## Supporting Information

Figure S1 **CD103^+^ -like and CD11b^+^ -type DCs induce antigen-specific CD8^+^ T cell responses upon transduction with Cav-NS1 R^0^ and Cav-VP7 R^0^.** CD103^+^ and CD11b^+^ -type DCs (2×10^4^) were cytometry-sorted from sheep #61 and pulsed overnight with Cav-NS1 R^0^ and Cav-VP7 R^0^ (BTV label), with Cav-null R^0^ (null label) or left alone (- label). Autologous BTV-immune CD8^+^ T cells (2×10^5^) were added to the wells (2–4 replicates) and the co-culture was pursued for 72 hours. Supernatants were harvested for IFNγ detection by ELISA. Statistical significance (paired Student t-test) is indicated by ** for p < 0.005 and * for p < 0.05.
(TIF)

Figure S2 **CAV2 triggers skin-migrated DC activation.** (A) lymph DCs from 2 different sheep (⧫ #70 and ▴ #66) were isolated by negative selection and they were cultured alone or with 50 TCID_50_/cell Cav-null R^0^ for 3, 16 and 24 hours. Cells were lysed for RNA extraction and real time RT-PCR. The ratio of the GAPDH-normalized cytokine mRNA signals in Cav-null R^0^ -stimulated versus non stimulated cultures was calculated. (B) After a 36 hour infection with 50 TCID_50_/cell Cav-null R^0^, LDL cells from sheep #81 (black bar) and sheep #70 (grey bar) were co-labeled for detection of the CD1b (FL-1) together with the CD80, CD86, CD40 and MHC class 2 molecules (FL-2). The ratio of the mean fluorescence intensity (MFI) corresponding to CD80, CD86, CD40 and MHC class 2 expression on the gated CD1b^+^ cells from cultures with Cav-null R^0^ versus from mock cultures is reported. Isotype control staining of the control and Cav-null R^0^ -activated cell cultures were identical (not shown). (C) Cytometry profile of CD80 and CD86 expression induced by Cav-null R^0^ on CD1b^+^ cells (infected CD1b^+^ cells, thick black line; mock cultured CD1b^+^ cells, grey filling). The increase in CD80/CD86 expression concerns the whole CD1b^+^ population.
(TIF)

Table S1 Up-regulated genes induced by CAV2 vector in skin-migrated DC subsets.
(DOC)

Table S2 Down-modulated genes by CAV2 vector in skin-migrated DC subsets.
(DOC)
