# Supplementary figures and images for: Canine Recombinant Adenovirus Vector Induces an Immunogenicity-Related Gene Expression Profile in Skin-Migrated CD11b+ -Type DCs
Source: PLoS One. 2012 Dec 26;7(12):e52513. doi: 10.1371/journal.pone.0052513 (PMC3530480; doi:10.1371/journal.pone.0052513)

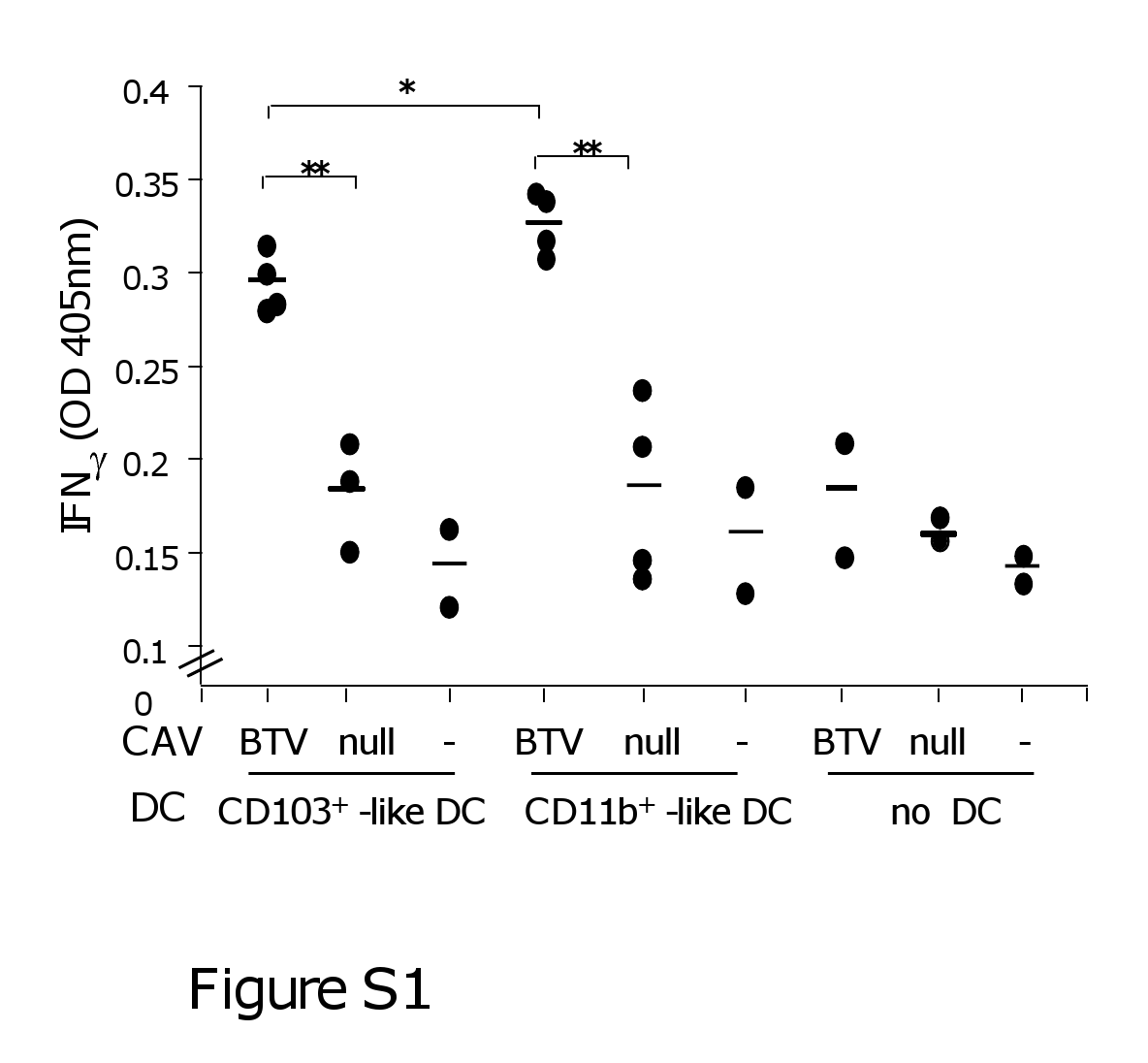

Supplement: Figure S1 — CD103+ -like and CD11b+ -type DCs induce antigen-specific CD8+ T cell responses upon transduction with Cav-NS1 R0 and Cav-VP7 R0. CD103+ and CD11b+ -type DCs (2×10^4^) were cytometry-sorted from sheep #61 and pulsed overnight with Cav-NS1 R0 and Cav-VP7 R0 (BTV label), with Cav-null R0 (null label) or left alone (- label). Autologous BTV-immune CD8+ T cells (2×10^5^) were added to the wells (2–4 replicates) and the co-culture was pursued for 72 hours. Supernatants were harvested for IFNγ detection by ELISA. Statistical significance (paired Student t-test) is indicated by ** for p < 0.005 and * for p < 0.05. (TIF) [file pone.0052513.s001.tif]

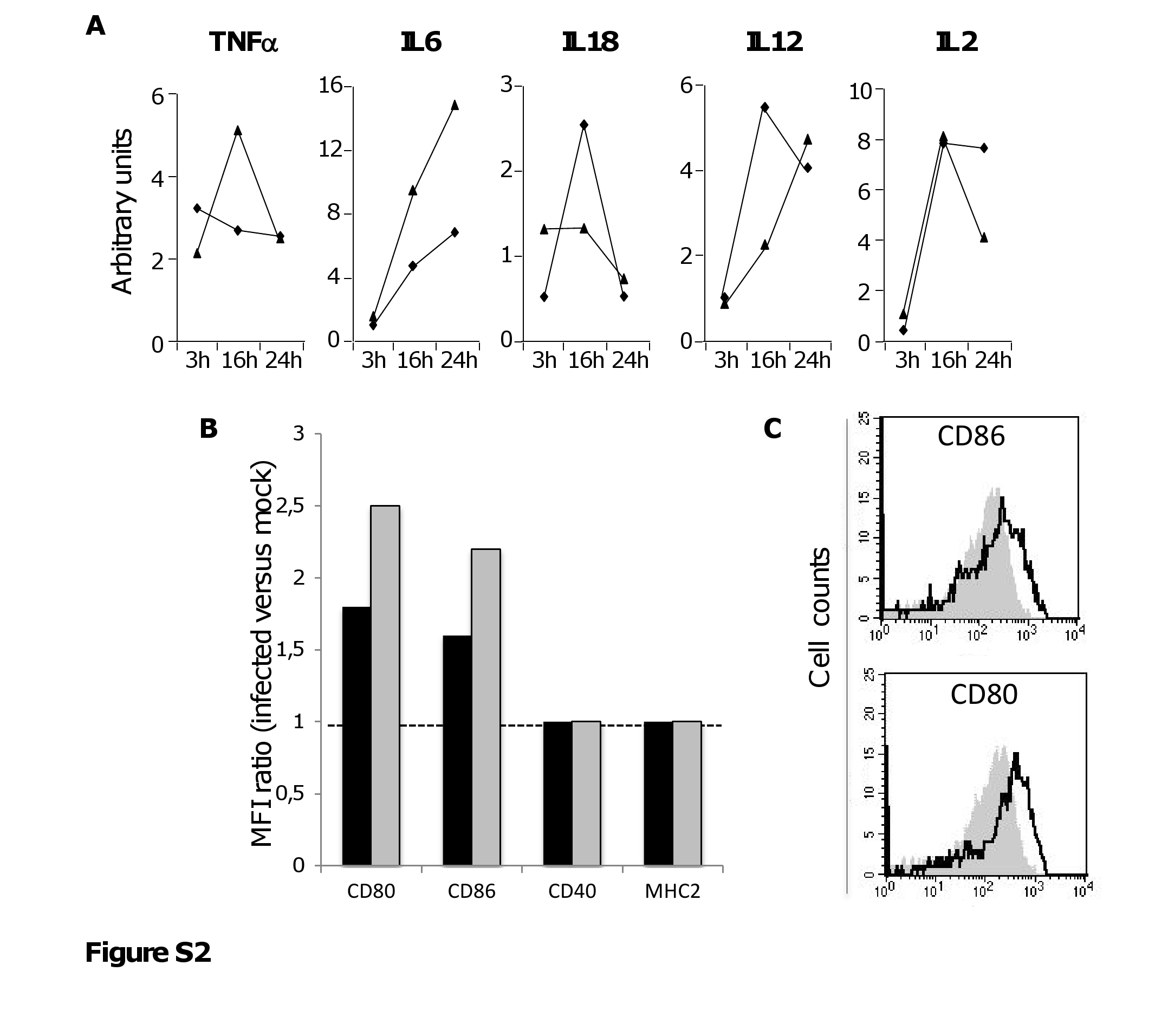

Supplement: Figure S2 — CAV2 triggers skin-migrated DC activation. (A) lymph DCs from 2 different sheep (⧫ #70 and ▴ #66) were isolated by negative selection and they were cultured alone or with 50 TCID50/cell Cav-null R0 for 3, 16 and 24 hours. Cells were lysed for RNA extraction and real time RT-PCR. The ratio of the GAPDH-normalized cytokine mRNA signals in Cav-null R0 -stimulated versus non stimulated cultures was calculated. (B) After a 36 hour infection with 50 TCID50/cell Cav-null R0, LDL cells from sheep #81 (black bar) and sheep #70 (grey bar) were co-labeled for detection of the CD1b (FL-1) together with the CD80, CD86, CD40 and MHC class 2 molecules (FL-2). The ratio of the mean fluorescence intensity (MFI) corresponding to CD80, CD86, CD40 and MHC class 2 expression on the gated CD1b+ cells from cultures with Cav-null R0 versus from mock cultures is reported. Isotype control staining of the control and Cav-null R0 -activated cell cultures were identical (not shown). (C) Cytometry profile of CD80 and CD86 expression induced by Cav-null R0 on CD1b+ cells (infected CD1b+ cells, thick black line; mock cultured CD1b+ cells, grey filling). The increase in CD80/CD86 expression concerns the whole CD1b+ population. (TIF) [file pone.0052513.s002.tif]
