# Supplementary material for: Canine Recombinant Adenovirus Vector Induces an Immunogenicity-Related Gene Expression Profile in Skin-Migrated CD11b+ -Type DCs
Source: PLoS One. 2012 Dec 26;7(12):e52513. doi: 10.1371/journal.pone.0052513 (PMC3530480; doi:10.1371/journal.pone.0052513)
Supplement: Table S1 — Up-regulated genes induced by CAV2 vector in skin-migrated DC subsets. (DOC) [file pone.0052513.s003.doc]

**Table S1: Up-regulated genes induced by CAV2 vector in skin-migrated DC subsets**

| **Gene symbol** | **General identifier of gene name** |  | **CD11b^+^ -type** | | |  | **CD103^+^ -type** | | |  | |
| --- | --- | --- | --- | --- | --- | --- | --- | --- | --- | --- | --- |
|  |  |  | **Adj pValue** | **Fold** | **qRT-PCR** |  | **Adj pValue** | **Fold** | **qRT-PCR** |  | |
| MX2 | myxovirus (influenza virus) resistance 2 |  | 0.0005 | 80.2163 | - |  | 0.0588 | 12.2974 | - |  | |
| MX1* | myxovirus (influenza virus) resistance 1 |  | 0.0001 | 75.1086 | 12-149 |  | 0.0041 | 20.3501 | 33-179 |  | |
| EIF4E | eukaryotic translation initiation factor 4E |  | 0.0010 | 23.3713 | - |  | 0.0837 | 6.1192 | - |  | |
| ISG15 | Ubiquitin cross-reactive protein (Interferon-stimulated gene product 17) | | 0.0001 | 22.1684 | - |  | 0,0054 | 9,2853 | - |  | |
| Q2HJD9 | 28kD interferon responsive protein |  | 0.0016 | 16.1707 | - |  | 0.0716 | 5.9265 | - |  | |
| Fap | fibroblast activation protein, alpha |  | 0.0004 | 16.0192 | - |  | 0.0677 | 4.3042 | - |  | |
| IFI44 | interferon-induced protein 44 |  | 0.0079 | 15.8133 | - |  | 0.2485 | 5.7997 | - |  | |
| GCLC | glutamate-cysteine ligase catalytic subunit |  | 0.0051 | 15.7523 | - |  | 0.2643 | 4.7032 | - |  | |
| KIAA1618 | KIAA1618 |  | 0.0004 | 15.2466 | - |  | 0.0490 | 4.5650 | - |  | |
| CXCL10 | chemokine (C-X-C motif) ligand 10 |  | 0.0299 | 14.5446 | 2.8-20 |  | 0.6222 | 2.7771 | 1.5-40 |  | |
| XAF1 | XIAP-associated factor 1 |  | 0.0024 | 13,8799 | - |  | 0,1771 | 4,3709 | - |  | |
| TRIM5 | tripartite motif-containing 5 |  | 0,0022 | 12,4939 | - |  | 0,2372 | 3,4513 | - |  | |
| OAS2 | 2'-5'-oligoadenylate synthetase 2, 69/71kDa; |  | 0,0001 | 11,6513 | - |  | 0,0221 | 3,8328 | - |  | |
| LOC782527 | similar to tripartite motif protein 5 alpha |  | 0,0041 | 11,4235 | - |  | 0,3343 | 3,1153 | - |  | |
| Trank1 | tetratricopeptide repeat and ankyrin repeat containing 1 |  | 0,0023 | 10,7062 | - |  | 0,3176 | 2,7775 | - |  | |
| ZNFX1 | zinc finger, NFX1-type containing 1 |  | 0.0007 | 10.5141 | - |  | 0.0588 | 4.0641 | - |  | |
| EIF2AK2 | eukaryotic translation initiation factor 2-alpha kinase 2 |  | 0.0016 | 10.2271 | 13-25 |  | 0.0677 | 4.7496 | 6.44-21 |  | |
| IFIH1 | interferon induced with helicase C domain 1 |  | 0.0002 | 10.1363 | - |  | 0.0411 | 3.3174 | - |  | |
| TNF | Tumor necrosis factor Precursor (TNF-alpha) |  | 0.0001 | 9.0721 | 6.1-14.7 |  | 0.0861 | 2.4897 | 1.6-2.3 |  | |
| GBP5 | guanylate-binding protein 5 |  | 0.0103 | 8.5217 | - |  | 0.2485 | 4.4048 | - |  | |
| PARP14 | PARP14 protein Fragment |  | 0.0012 | 8.4909 | - |  | 0.0697 | 3.7359 | - |  | |
| Myof | myoferlin |  | 0.0084 | 8.4306 | - |  | 0.3532 | 3.0865 | - |  | |
| RNF213 | ring finger protein 213-like |  | 0.0042 | 8.3595 | - |  | 0.2380 | 3.3062 | - |  | |
| Pyhin1 | pyrin and HIN domain family, member 1 |  | 0.0006 | 7.9949 | - |  | 0.0697 | 3.1716 | - |  | |
| GNLY | granulysin |  | 0.0065 | 7.6934 | - |  | 0.0857 | 5.6140 | - |  | |
| IL27 | interleukin 27 |  | 0.0001 | 7.0694 | - |  | 0.0381 | 2.3879 | - |  | |
| UBA7 | ubiquitin-like modifier activating enzyme 7 |  | 0.0096 | 7.0160 | - |  | 0.4853 | 2.1995 | - |  | |
| CCL5 | chemokine (C-C motif) ligand 5 |  | 0.0016 | 6.9800 | - |  | 0.0411 | 4.1957 | - |  | |
| IFITM1 | interferon induced transmembrane protein 1 |  | 0.0090 | 6.6878 | - |  | 0.6481 | 1.6361 | - |  | |
| PARP9 | poly (ADP-ribose) polymerase family, member 9 |  | 0.0010 | 6.5434 | - |  | 0.0495 | 3.4096 | - |  | |
| EPSTI1 | epithelial stromal interaction 1 (breast) |  | 0.0004 | 6.4528 | - |  | 0.1384 | 2.2106 | - |  | |
| GBP3 | interferon-induced guanylate-binding protein 2-like |  | 0.0339 | 5.9930 | - |  | 0.7750 | 1.5591 | - |  | |
| ETS2 | Protein C-ets-2 |  | 0.0094 | 5.9324 | 2.68-14.2 |  | 0.4692 | 2.1002 | 2.25-9.5 |  | |
| NCAPG | non-SMC condensin I complex |  | 0.0008 | 5.9216 | - |  | 0.0206 | 3.8956 | - |  | |
| LOC781710 | hypothetical protein LOC781710 |  | 0.0035 | 5.7888 | - |  | 0.3919 | 1.9889 | - |  | |
| LOC511531 | Bos taurus interferon-induced guanylate-binding protein 2-like | | 0.0183 | 5.4595 | - |  | 0.7989 | 1.3756 | - |  |  |
| TRIM34 | tripartite motif-containing 6 and tripartite motif-containing 34 |  | 0.0066 | 5.2213 | - |  | 0.4184 | 2.0055 | - |  | |
| SAMD9 | sterile alpha motif domain containing 9 |  | 0.0101 | 5.2120 | - |  | 0.4957 | 1.9356 | - |  | |
| WARS | Tryptophanyl-tRNA synthetase |  | 0.0002 | 5.0456 | - |  | 0.0797 | 2.0955 | - |  | |
| PAF | KIAA0101 protein |  | 0.0135 | 5.0439 | - |  | 0.3479 | 2.7086 | - |  | |
| PML | similar to promyelocytic leukemia protein |  | 0.0018 | 4.9127 | - |  | 0.2238 | 2.1505 | - |  | |
| Tmprss2 | transmembrane protease, serine 2 |  | 0.0023 | 4.9016 | - |  | 0.6122 | 1.3890 | - |  | |
| NDC80 | NDC80 homolog, kinetochore complex component (S. cerevisiae) |  | 0.0009 | 4.8132 | - |  | 0.0736 | 2.4791 | - |  | |
| B2MWQ2 | MHC class I-related protein Fragment |  | 0.0037 | 4.7336 | - |  | 0.2372 | 2.3447 | - |  | |
| CIT | rho-interacting, serine/threonine kinase 21 |  | 0.0023 | 4.7058 | - |  | 0.1426 | 2.4931 | - |  | |
| ACADM | Medium-chain specific acyl-CoA dehydrogenase |  | 0.0090 | 4.5941 | - |  | 0.4164 | 2.0139 | - |  | |
| sds | similar to serine dehydratase |  | 0.0059 | 4.4880 | - |  | 0.6836 | 1.3749 | - |  | |
| FTSJD1 | FtsJ methyltransferase domain containing 1 |  | 0.0071 | 4.4015 | - |  | 0.3434 | 2.1796 | - |  | |
| CD8B | CD8b antigen |  | 0.0098 | 4.3127 | - |  | 0.7617 | 1.3173 | - |  | |
| ADAR | Double-stranded RNA-specific adenosine deaminase (DRADA) |  | 0.0030 | 4.2907 | - |  | 0.1631 | 2.4077 | - |  | |
| REG1 | Regakine-1 Precursor |  | 0.0250 | 4.2283 | - |  | 0.8713 | 1.2268 | - |  | |
| NFE2L3 | nuclear factor (erythroid-derived 2)-like 3 |  | 0.0118 | 4.2057 | - |  | 0.4642 | 1.9208 | - |  | |
| PNPT1 | polyribonucleotide nucleotidyltransferase 1 |  | 0.0192 | 4.1709 | - |  | 0.4733 | 2.0468 | - |  | |
| CFB | complement factor B |  | 0.0294 | 4.1602 | - |  | 0.7554 | 1.4355 | - |  | |
| GM2A | GM2 ganglioside activator |  | 0.0167 | 4.1585 | - |  | 0.5163 | 1.8425 | - |  | |
| ATXN1 | ataxin 3 |  | 0.0190 | 4.1532 | - |  | 0.5512 | 1.8019 | - |  | |
| dusp5 | dual specificity phosphatase 5 |  | 0.0124 | 4.1321 | - |  | 0.4583 | 1.9366 | - |  | |
| KIF2C | kinesin family member 2C |  | 0.0069 | 4.1297 | - |  | 0.3658 | 2.0193 | - |  | |
| PTGR1 | prostaglandin reductase 1 |  | 0.0391 | 3.9377 | - |  | 0.6516 | 1.6895 | - |  | |
| AURKB | aurora kinase B |  | 0.0041 | 3.8074 | - |  | 0.2014 | 2.2291 | - |  | |
| DAXX | death-associated protein 6 |  | 0.0037 | 3.7808 | - |  | 0.2581 | 2.0163 | - |  | |
| Grsf1 | G-rich RNA sequence binding factor 1 |  | 0.0030 | 3.7804 | - |  | 0.2114 | 2.0657 | - |  | |
| FBXO33 | F-box protein 33 |  | 0.0066 | 3.7778 | - |  | 0.3827 | 1.8482 | - |  | |
| KCNJ2 | potassium inwardly-rectifying channel, subfamily J, member 2 |  | 0.0184 | 3.7414 | - |  | 0.3715 | 2.2842 | - |  | |
| IFI35 | interferon-induced protein 35 |  | 0.0099 | 3.7219 | - |  | 0.4689 | 1.7606 | - |  | |
| ATXN3 | ataxin 3 |  | 0.0185 | 3.6741 | - |  | 0.4957 | 1.8381 | - |  | |
| LAYN | layilin |  | 0.0251 | 3.6275 | - |  | 0.7484 | 1.3840 | - |  | |
| FOXS1 | forkhead box S1 |  | 0.0316 | 3.5809 | - |  | 0.5356 | 1.8584 | - |  | |
| KCNK1 | potassium channel, subfamily K, member 1 |  | 0.0183 | 3.5645 | - |  | 0.5835 | 1.5964 | - |  | |
| GSR | Bos taurus misc_RNA (LOC784358) glutathione reductase |  | 0.0021 | 3.5470 | - |  | 0.1704 | 2.0074 | - |  | |
| GTF2E1 | General transcription factor IIE subunit 1 |  | 0.0081 | 3.5457 | - |  | 0.4076 | 1.7787 | - |  | |
| CTSL2 | Cathepsin L1 Precursor |  | 0.0331 | 3.5451 | - |  | 0.5824 | 1.7411 | - |  | |
| STAT1 | signal transducer and activator of transcription 1 |  | 0.0078 | 3.5279 | 1.8-2.8 |  | 0.3436 | 1.9576 | 1.5-3 |  | |
| SP110 | SP110 nuclear body protein |  | 0.0007 | 3.5260 | - |  | 0.2343 | 1.6498 | - |  | |
| LOC786906 | cyclin A2; similar to Cyclin A2 |  | 0.0103 | 3.5237 | - |  | 0.3791 | 1.9536 | - |  | |
| cyp2j2 | cytochrome P450, family 2, subfamily J, polypeptide 2 |  | 0.0112 | 3.5216 | - |  | 0.5356 | 1.5959 | - |  | |
| XRN2 | XRN2 protein Fragment |  | 0.0113 | 3.4479 | - |  | 0.4577 | 1.7538 | - |  | |
| fam54a | DUF729 domain containing 1 |  | 0.0001 | 3.4314 | - |  | 0.0032 | 2.3433 | - |  | |
| SLAMF7 | SLAM family member 7 |  | 0.0005 | 3.4175 | - |  | 0.2372 | 1.5742 | - |  | |
| OGFR | opioid growth factor receptor |  | 0.0226 | 3.4069 | - |  | 0.6000 | 1.5886 | - |  | |
| TTK | TTK protein kinase |  | 0.0003 | 3.4002 | - |  | 0.0158 | 2.2808 | - |  | |
| DKK3 | dickkopf homolog 3 (Xenopus laevis) |  | 0.0007 | 3.3973 | - |  | 0.0762 | 1.9703 | - |  | |
| MYCBP | c-myc binding protein |  | 0.0183 | 3.3735 | - |  | 0.4290 | 1.9266 | - |  | |
| NCAPH | non-SMC condensin I complex, subunit H (NCAPH) |  | 0.0004 | 3.3606 | - |  | 0.0411 | 2.0352 | - |  | |
| diaph3 | diaphanous homolog 3 (Drosophila) |  | 0.0001 | 3.3566 | - |  | 0.0019 | 2.2395 | - |  | |
| MGC139164 | hypothetical LOC509649 |  | 0.0288 | 3.3183 | - |  | 0.7087 | 1.4281 | - |  | |
| CDCA8 | cell division cycle associated 8 |  | 0.0035 | 3.2650 | - |  | 0.2343 | 1.9081 | - |  | |
| PPP1R1A | protein phosphatase 1, regulatory (inhibitor) subunit 1A |  | 0.0056 | 3.2644 | - |  | 0.6663 | 1.2953 | - |  | |
| LAT | LAT protein |  | 0.0001 | 3.2426 | - |  | 0.0206 | 1.8943 | - |  | |
| COX7A1 | Cytochrome c oxidase polypeptide 7A1 |  | 0.0035 | 3.2154 | - |  | 0.3452 | 1.6734 | - |  | |
| EDN1 | Endothelin-1 Precursor (ET-1) |  | 0.0055 | 3.2142 | - |  | 0.2936 | 1.8851 | - |  | |
| PSMF1 | proteasome (prosome, macropain) inhibitor subunit 1 (PI31) |  | 0.0094 | 3.1778 | - |  | 0.5738 | 1.4513 | - |  | |
| PLK2 | peroxiredoxin 1 |  | 0.0031 | 3.1721 | - |  | 0.2643 | 1.7903 | - |  | |
| Ckap2 | cytoskeleton associated protein 2 |  | 0.0004 | 3.1687 | - |  | 0.0123 | 2.3981 | - |  | |
| CKS2 | Cyclin-dependent kinases regulatory subunit 2 (CKS-2) |  | 0.0005 | 3.1458 | - |  | 0.0245 | 2.1445 | - |  | |
| TNFSF13B | tumor necrosis factor (ligand) superfamily, member 13b |  | 0.0007 | 3.1203 | 2-3.5 |  | 0.1099 | 1.7652 | 1.5-2.5 |  | |
| AIDA | axin interactor, dorsalization associated |  | 0.0066 | 3.1130 | - |  | 0.4957 | 1.4988 | - |  | |
| Dnaja1 | DnaJ (Hsp40) homolog, subfamily A, member 1 |  | 0.0066 | 3.0931 | - |  | 0.3729 | 1.7138 | - |  | |
| Fam13a1 | family with sequence similarity 13, member A1 |  | 0.0149 | 3.0817 | - |  | 0.3527 | 2.0121 | - |  | |
| CDCA5 | cell division cycle associated 5 |  | 0.0025 | 3.0452 | - |  | 0.0677 | 2.2726 | - |  | |
| ASF1B | Histone chaperone ASF1B (Anti-silencing function protein 1 homolog B) | | 0.0051 | 3.0424 | - |  | 0.1561 | 2.1303 |  |  | |
| C5orf32 | UPF0467 protein C5orf32 homolog |  | 0.0130 | 3.0306 | - |  | 0.4856 | 1.6227 | - |  | |
| SDC4 | syndecan 4-like |  | 0.0040 | 3.0288 | - |  | 0.5582 | 1.3506 | - |  | |
| MIA3 | melanoma inhibitory activity family, member 3 |  | 0.0140 | 3.0273 | - |  | 0.4704 | 1.6708 | - |  | |
| CASP5 | Caspase-13 Precursor (CASP-13) |  | 0.0174 | 3.0145 | - |  | 0.4403 | 1.7812 | - |  | |
| LGALS3BP | Galectin-3-binding protein Precursor |  | 0.0094 | 2.9849 | - |  | 0.6390 | 1.3464 | - |  | |
| PELI1 | pellino homolog 1 (Drosophila) |  | 0.0014 | 2.9811 | - |  | 0.1777 | 1.7154 | - |  | |
| Pdzk1 | PDZ domain containing 1 |  | 0.0001 | 2.9403 | - |  | 0.0009 | 3.1113 | - |  | |
| USP25 | ubiquitin specific peptidase 25 |  | 0.0090 | 2.9248 | - |  | 0.4186 | 1.6305 | - |  | |
| CDC2 | Cell division control protein 2 homolog |  | 0.0044 | 2.9245 | - |  | 0.2776 | 1.7731 | - |  | |
| VCPIP1 | valosin containing protein |  | 0.0064 | 2.8892 | - |  | 0.3800 | 1.6329 | - |  | |
| C13orf15 | hypothetical protein LOC614348 |  | 0.0224 | 2.8721 | - |  | 0.6982 | 1.3542 | - |  | |
| SMAD1 | SMAD family member 1 |  | 0.0177 | 2.8453 | - |  | 0.5368 | 1.5451 | - |  | |
| STAT2 | signal transducer and activator of transcription 2, 113kDa |  | 0.0189 | 2.8430 | - |  | 0.5452 | 1.5480 | - |  | |
| C18orf24 | hypothetical protein LOC507703 |  | 0.0053 | 2.8371 | - |  | 0.1746 | 2.0117 | - |  | |
| UBE2J1 | ubiquitin-conjugating enzyme E2, J1 |  | 0.0139 | 2.8370 | - |  | 0.4692 | 1.6246 | - |  | |
| ESPL1 | extra spindle pole bodies homolog 1 (S. cerevisiae) |  | 0.0001 | 2.8336 | - |  | 0.0041 | 2.0541 | - |  | |
| MARCKSL1 | Bos taurus MARCKS-like 1 (MARCKSL1) |  | 0.0024 | 2.7962 | - |  | 0.2610 | 1.6456 | - |  | |
| UBE2C | Bos taurus ubiquitin-conjugating enzyme E2C (UBE2C), mRNA |  | 0.0154 | 2.7862 | - |  | 0.3742 | 1.8398 | - |  | |
| CCL4 | C-C motif chemokine 4 Precursor (Small-inducible cytokine A4) |  | 0.0094 | 2.7821 |  |  | 0.3779 | 1.6983 |  |  | |
| NAMPT | nicotinamide phosphoribosyltransferase |  | 0.0082 | 2.7725 | - |  | 0.4164 | 1.5747 | - |  | |
| BIRC5 | baculoviral IAP repeat-containing 5 |  | 0.0046 | 2.7697 | - |  | 0.1074 | 2.1126 | - |  | |
| IL6 | Interleukin-6 Precursor (IL-6) |  | 0.0022 | 2.7570 | - |  | 0.2936 | 1.5676 | - |  | |
| ALOX5AP | Arachidonate 5-lipoxygenase-activating protein ] |  | 0.0272 | 2.7540 | - |  | 0.7978 | 1.2360 | - |  | |
| BUB1 | BUB1 budding uninhibited by benzimidazoles 1 homolog |  | 0.0026 | 2.7277 | - |  | 0.0411 | 2.3029 | - |  | |
| DBR1 | debranching enzyme homolog 1 (S. cerevisiae) |  | 0.0173 | 2.7010 | - |  | 0.9730 | 1.0319 | - |  | |
| ANPEP | alanyl (membrane) aminopeptidase |  | 0.0284 | 2.6911 | - |  | 0.5005 | 1.6597 | - |  | |
| Usp18 | ubiquitin specific peptidase 18 |  | 0.0155 | 2.6795 | - |  | 0.5195 | 1.5066 | - |  | |
| TRAFD1 | TRAF-type zinc finger domain containing 1 |  | 0.0130 | 2.6748 | - |  | 0.5605 | 1.4301 | - |  | |
| CDKN2AIP | CDKN2A interacting protein |  | 0.0221 | 2.6636 | - |  | 0.4745 | 1.6640 | - |  | |
| MOV10 | Mov10, Moloney leukemia virus 10, homolog |  | 0.0116 | 2.6620 | - |  | 0.6478 | 1.3155 | - |  | |
| MAT2B | methionine adenosyltransferase II, beta |  | 0.0088 | 2.6581 | - |  | 0.4197 | 1.5526 | - |  | |
| TRIM26 | tripartite motif-containing 26 |  | 0.0099 | 2.6397 | - |  | 0.4335 | 1.5610 | - |  | |
| AQP4 | Aquaporin-4 (AQP-4) |  | 0.0263 | 2.6326 | - |  | 0.8494 | 1.1698 | - |  | |
| LOC788943 | X-box binding protein 1 isoform XBP1(U)-like (LOC788943), |  | 0.0313 | 2.5915 | - |  | 0.7691 | 1.2656 | - |  | |
| RNF114 | RING finger protein 114 (Zinc finger protein 313) |  | 0.0109 | 2.5717 | - |  | 0.5775 | 1.3704 | - |  | |
| PDGFRL | Platelet-derived growth factor receptor-like protein Precursor |  | 0.0016 | 2.5694 | - |  | 0.3494 | 1.4149 | - |  | |
| CD80 | CD80/B7-1 transmembrane form |  | 0.0022 | 2.5665 | - |  | 0.4405 | 1.3479 | - |  | |
| CCND3 | cyclin D3, |  | 0.0339 | 2.5650 | - |  | 0.7128 | 1.3335 | - |  | |
| glrx5 | glutaredoxin 5 |  | 0.0033 | 2.5243 | - |  | 0.2936 | 1.5673 | - |  | |
| LOC100037692 | X box-binding protein active isoform |  | 0.0333 | 2.5204 | - |  | 0.8296 | 1.1953 | - |  | |
| ZC3HAV1 | CCCH-type zinc finger antiviral protein-like (ZC3HAV1), |  | 0.0005 | 2.5189 | - |  | 0.1140 | 1.5471 | - |  | |
| DNAJC13 | DnaJ (Hsp40) homolog, subfamily C, member 13 (DNAJC13), |  | 0.0138 | 2.5082 | - |  | 0.4191 | 1.6012 | - |  | |
| cpd | carboxypeptidase D |  | 0.0097 | 2.4907 | - |  | 0.5163 | 1.4069 | - |  | |
| RDM1 | RAD52 motif 1 |  | 0.0237 | 2.4840 | - |  | 0.1746 | 2.4689 | - |  | |
| RBBP6 | retinoblastoma binding protein 6 |  | 0.0120 | 2.4689 | - |  | 0.3791 | 1.6459 | - |  | |
| slc1a4 | solute carrier family1 (glutamate/neutral amino acid transportermember 4) | | 0.0388 | 2.4603 |  |  | 0.6881 | 1.3617 | - |  | |
| CCNB2 | G2/mitotic-specific cyclin-B2 |  | 0.0005 | 2.4500 | - |  | 0.0381 | 1.7747 | - |  | |
| PRDX1 | peroxiredoxin 1 |  | 0.0019 | 2.4443 | - |  | 0.1746 | 1.6124 | - |  | |
| DHX29 | DEAH (Asp-Glu-Ala-His) box polypeptide 29 |  | 0.0309 | 2.4222 | - |  | 0.8036 | 1.2062 | - |  | |
| TOR1AIP2 | torsin A interacting protein 2 (TOR1AIP2), transcript variant 2, |  | 0.0361 | 2.4221 | - |  | 0.6006 | 1.4534 | - |  | |
| CKAP2L | cytoskeleton associated protein 2-like (CKAP2L), |  | 0.0017 | 2.4208 | - |  | 0.1962 | 1.5623 | - |  | |
| TACC3 | transforming, acidic coiled-coil containing protein 3 |  | 0.0001 | 2.4038 | - |  | 0.0013 | 2.2251 | - |  | |
| csf1 | colony stimulating factor 1 (macrophage) |  | 0.0071 | 2.3968 | - |  | 0.4598 | 1.4175 | - |  | |
| ATAD1 | ATAD1 protein Fragment |  | 0.0124 | 2.3908 | - |  | 0.5745 | 1.3540 | - |  | |
| PARP10 | poly (ADP-ribose) polymerase family, member 100 |  | 0.0362 | 2.3854 | - |  | 0.4957 | 1.6213 | - |  | |
| IRF3 | Interferon regulatory factor 3 (IRF-3) |  | 0.0216 | 2.3774 | - |  | 0.6666 | 1.3112 | - |  | |
| ATF3 | Cyclic AMP-dependent transcription factor ATF-3 |  | 0.0001 | 2.3661 | - |  | 0.0163 | 1.5347 | - |  | |
| TXNIP | thioredoxin interacting protein |  | 0.0001 | 2.3598 | - |  | 0.0032 | 1.9216 | - |  | |
| ABTB2 | ankyrin repeat and BTB (POZ) domain containing 2 |  | 0.0060 | 2.3430 | - |  | 0.4995 | 1.3370 | - |  | |
| IGHV3-6 | IGHM protein |  | 0.0193 | 2.3334 | - |  | 0.4764 | 1.5257 | - |  | |
| Setx | similar to SETX protein |  | 0.0205 | 2.3308 | - |  | 0.4988 | 1.4951 | - |  | |
| PIM2 | PIM2 protein Fragment |  | 0.0107 | 2.3302 | - |  | 0.3800 | 1.5701 | - |  | |
| CKS1B | CDC28 protein kinase regulatory subunit 1B |  | 0.0036 | 2.3230 | - |  | 0.0356 | 2.1940 | - |  | |
| WDR51A | WD repeat-containing protein 51A |  | 0.0090 | 2.3203 | - |  | 0.4604 | 1.4249 | - |  | |
| CCRK | cell cycle related kinase |  | 0.0112 | 2.3104 | - |  | 0.5645 | 1.3356 | - |  | |
| TBC1D8B | TBC1 domain family, member 8B |  | 0.0258 | 2.3021 | - |  | 0.3504 | 1.8225 | - |  | |
| DLGAP5 | discs large homolog 7 |  | 0.0189 | 2.3020 | - |  | 0.2326 | 2.0175 | - |  | |
| JAG1 | jagged 1 (Alagille syndrome) |  | 0.0014 | 2.2966 | - |  | 0.1114 | 1.5928 | - |  | |
| C3orf1 | hypothetical protein LOC507206 |  | 0.0205 | 2.2913 | - |  | 0.8673 | 1.1210 | - |  | |
| KCNN3 | potassium intermediate/small conductance calcium-activated channel, subfamily N, member 3 | | 0.0096 | 2.2826 | - |  | 0.2519 | 1.7446 | - |  | |
| INPP5B | inositol polyphosphate-5-phosphatase, 75kDa |  | 0.0049 | 2.2745 | - |  | 0.3929 | 1.4078 | - |  | |
| Fancd2 | Fanconi anemia, complementation group D2 |  | 0.0028 | 2.2678 | - |  | 0.1375 | 1.6637 | - |  | |
| CD200 | CD200 molecule |  | 0.0051 | 2.2616 | - |  | 0.3494 | 1.4693 | - |  | |
| SPSB1 | SPRY domain-containing SOCS box protein 1 (SSB-1) |  | 0.0042 | 2.2573 | - |  | 0.3929 | 1.3885 | - |  | |
| FLT1 | VEGF-receptor (Flt-receptor) Fragment |  | 0.0132 | 2.2412 | - |  | 0.8652 | 1.1064 | - |  | |
| PSORS1C2 | psoriasis susceptibility 1 candidate 2 |  | 0.0184 | 2.2300 | - |  | 0.8515 | 1.1242 | - |  | |
| Klf4 | Kruppel-like factor 4 (gut) |  | 0.0030 | 2.2205 | - |  | 0.4088 | 1.3342 | - |  | |
| TP53INP2 | tumor protein p53 inducible nuclear protein 2 |  | 0.0020 | 2.2191 | - |  | 0.0716 | 1.7222 | - |  | |
| AURKA | Serine/threonine-protein kinase 6 |  | 0.0032 | 2.2163 | - |  | 0.1456 | 1.6471 | - |  | |
| RNF19B | ring finger protein 19B |  | 0.0084 | 2.2097 | - |  | 0.4692 | 1.3792 | - |  | |
| LMNA | lamin A/C |  | 0.0436 | 2.2080 | - |  | 0.9225 | 1.0893 | - |  | |
| HMGB2 | high-mobility group box 2 |  | 0.0051 | 2.2063 | - |  | 0.1771 | 1.6804 | - |  | |
| CFLAR | CASP8 and FADD-like apoptosis regulator |  | 0.0023 | 2.2042 | - |  | 0.3465 | 1.3760 | - |  | |
| TYSY | Thymidylate synthase (TSase) |  | 0.0014 | 2.1970 | - |  | 0.0221 | 1.8780 | - |  | |
| STK17A | serine/threonine kinase 17a |  | 0.0116 | 2.1961 | - |  | 0.4971 | 1.3825 | - |  | |
| SGMS2 | sterile alpha motif domain containing 8-like |  | 0.0037 | 2.1881 | - |  | 0.9177 | 1.0481 | - |  | |
| HSPA1A | heat shock 7kDa protein 2 |  | 0.0200 | 2.1880 | - |  | 0.3543 | 1.6889 | - |  | |
| DNAJC7 | DNAJC7 protein Fragment |  | 0.0312 | 2.1849 | - |  | 0.5741 | 1.4121 | - |  | |
| CXCR3 | C-X-C chemokine receptor type 3 |  | 0.0228 | 2.1741 | - |  | 0.7664 | 0.8355 | - |  | |
| PARP12 | poly (ADP-ribose) polymerase family, member 12 (PARP12) |  | 0.0322 | 2.1571 | - |  | 0.5770 | 1.4042 | - |  | |
| MBP | myelin basic protein |  | 0.0058 | 2.1567 | - |  | 0.0155 | 2.5377 | - |  | |
| TIFA | hypothetical protein LOC783855 |  | 0.0489 | 2.1498 | - |  | 0.6753 | 1.3370 | - |  | |
| CYP51A1 | Cytochrome P450 51A1 |  | 0.0002 | 2.1497 | - |  | 0.0212 | 1.5846 | - |  | |
| LGALS13 | similar to galectin 15 |  | 0.0408 | 2.1449 | - |  | 0.6931 | 1.2982 | - |  | |
| CRYBB3 | Beta-crystallin B3 |  | 0.0193 | 2.1440 | - |  | 0.4226 | 1.5257 | - |  | |
| CENPE | centromere protein E |  | 0.0037 | 2.1424 | - |  | 0.0411 | 1.9680 | - |  | |
| xpo1 | exportin 1 (CRM1 homolog, yeast) |  | 0.0014 | 2.1385 | - |  | 0.0890 | 1.5652 | - |  | |
| psmA2 | similar to Proteasome subunit alpha type-2 |  | 0.0201 | 2.1348 | - |  | 0.5974 | 1.3226 | - |  | |
| LOC100141030 | similar to nuclear antigen Sp100 |  | 0.0058 | 2.1225 | - |  | 0.6215 | 1.2057 | - |  | |
| PRDM1 | PR domain containing 1, with ZNF domain |  | 0.0051 | 2.1214 | - |  | 0.3292 | 1.4502 | - |  | |
| PLK3 | polo-like kinase 3 |  | 0.0196 | 2.1166 | - |  | 0.4692 | 1.4636 | - |  | |
| AP1AR | hypothetical LOC533664, |  | 0.0192 | 2.1046 | - |  | 0.8814 | 1.0969 | - |  | |
| PCGF5 | polycomb group ring finger 5 |  | 0.0161 | 2.1041 | - |  | 0.6709 | 1.2353 | - |  | |
| PLAUR | Urokinase plasminogen activator surface receptor Precursor |  | 0.0069 | 2.1008 | - |  | 0.3815 | 1.4202 | - |  | |
| SHISA5 | Protein shisa-5 Precursor (Scotin) |  | 0.0397 | 2.0902 | - |  | 0.7731 | 1.2125 | - |  | |
| GCG | Glucagon Precursor |  | 0.0220 | 2.0899 | - |  | 0.8660 | 0.9011 | - |  | |
| TNFAIP8 | tumor necrosis factor, alpha-induced protein 8 |  | 0.0096 | 2.0852 | - |  | 0.5172 | 1.3141 | - |  | |
| AC104852.1 | Serine/threonine-protein kinase PLK1 |  | 0.0093 | 2.0850 | - |  | 0.3362 | 1.5189 | - |  | |
| TOM1L1 | target of myb1 (chicken)-like 1 |  | 0.0168 | 2.0845 | - |  | 0.7217 | 1.1988 | - |  | |
| arrdc4 | arrestin domain containing 4 |  | 0.0369 | 2.0796 | - |  | 0.3715 | 1.7363 | - |  | |
| SERTAD1 | SERTA domain containing 1 |  | 0.0066 | 2.0711 | - |  | 0.4419 | 1.3426 | - |  | |
| LOC784451 | similar to C-type lectin domain family 2, member h |  | 0.0365 | 2.0694 | - |  | 0.5827 | 1.3815 | - |  | |
| IGSF8 | immunoglobulin superfamily, member 8 |  | 0.0336 | 2.0682 | - |  | 0.8555 | 1.1293 | - |  | |
| LOC507055 | Bos taurus guanylate binding protein 4-like |  | 0.0178 | 2.0673 | - |  | 0.3203 | 1.6683 | - |  | |
| NEK2 | NIMA (never in mitosis gene a)-related kinase 2 |  | 0.0184 | 2.0667 | - |  | 0.2372 | 1.8196 | - |  | |
| cbr4 | carbonyl reductase 4 |  | 0.0095 | 2.0667 | - |  | 0.4091 | 1.4134 | - |  | |
| TCEAL1 | Transcription elongation factor A protein-like 1 |  | 0.0361 | 2.0631 | - |  | 0.6369 | 1.3261 | - |  | |
| DCK | Deoxycytidine kinase (dCK)(EC 2.7.1.74) |  | 0.0177 | 2.0631 | - |  | 0.2581 | 1.7611 | - |  | |
| RAD54L | RAD54-like protein |  | 0.0030 | 2.0556 | - |  | 0.2372 | 1.4603 | - |  | |
| RBMS1 | RNA binding motif, single stranded interacting protein 1 |  | 0.0112 | 2.0347 | - |  | 0.6668 | 1.2052 | - |  | |
| TTF2 | transcription termination factor, RNA polymerase II |  | 0.0026 | 2.0059 | - |  | 0.1099 | 1.5657 | - |  | |
| *CASP1*** | *Caspase-1 Fragment* |  | *0.0666* | *2.7054* | *1.75-6.28* |  | *0.8544* | *1.2216* | *1.1-4.25* |  | |
| *IL8*** | *Interleukin-8* |  | *0.0168* | *1.9932* | *1.3-2* |  | *0.3212* | *1.6323* | *1.4-2.6* |  | |
| *C1QB*** | *Complement component 1, q subcomponent, B chain* |  | *0.0719* | *2.2784* |  |  | *0.6777* | *1.4168* |  |  | |
| *IRF1*** | *Interferon Regulatory factor 1* |  | *0.0101* | *1.7091* | *1.1-1.9* |  | *0.4813* | *1.2693* | *1.25-1.9* |  | |
| *CCR7*** | *CC-Chemokin receptor 7* |  | *0.0060* | *1.7299* | *1.3-1.9* |  | *0.2414* | *1.4032* | *1.1-1.6* |  | |
| *IL12p40*** | *Interleukin-12* |  | *0.0461* | *1.9657* | *1.5-2.4* |  | *0.6409* | *1.3996* | *1.4-2.1* |  | |
| IRF7*** | *Interferon Regulatory factor 7* |  |  |  | 1.4-9.2 |  |  |  | 6.2-117 |  | |

In grey: gene significantly up-regulated by CAV2 vector (p<0.05 (LIMMA analysis) and fold >2) both in CD103^+^ -type and CD11b^+^ -type DCs

** Gene not selected as up-regulated by CAV2 vector but close to be selected and checked by qRT-PCR

*** Gene not present on the array. P < 0.05 (t-test) in the CD11b^+^ -type DCs
