# Supplementary material for: Canine Recombinant Adenovirus Vector Induces an Immunogenicity-Related Gene Expression Profile in Skin-Migrated CD11b+ -Type DCs
Source: PLoS One. 2012 Dec 26;7(12):e52513. doi: 10.1371/journal.pone.0052513 (PMC3530480; doi:10.1371/journal.pone.0052513)
Supplement: Table S2 — Down-modulated genes by CAV2 vector in skin-migrated DC subsets. (DOC) [file pone.0052513.s004.doc]

**Table S**2: Down-modulated genes by CAV2 vector in skin-migrated DC subsets

| **Gene symbol** | **General identifier of gene name** | **CD11b^+^ -type** | |  | **CD103^+^ -type** | |
| --- | --- | --- | --- | --- | --- | --- |
|  |  | **Adj pValue** | **Fold** |  | **Adj pValue** | **Fold** |
| VPS52 | Vacuolar protein sorting 52 homolog (S. cerevisiae) | 0.0208 | 0.4970 |  | 0.3929 | 0.7955 |
| SAMHD1 | SAM domain and HD domain 1 | 0.0052 | 0.4970 |  | 0.5424 | 0.8072 |
| MT2 | Metallothionein-2 (MT-2) | 0.0032 | 0.4961 |  | 0.3090 | 0.7207 |
| RAB7B | Ras-related protein Rab-7b | 0.0051 | 0.4866 |  | 0.3207 | 0.6963 |
| SLC2A4 | Solute carrier family 2. facilitated glucose transporter member 4 | 0.0344 | 0.4833 |  | 0.7961 | 1.1779 |
| ST3GAL2 | ST3 beta-galactoside alpha-2.3-sialyltransferase 2 | 0.0189 | 0.5124 |  | 0.6879 | 0.8248 |
| MOXD1 | monooxygenase. DBH-like 1 | 0.0093 | 0.4745 |  | 0.3779 | 0.6803 |
| MBD4 | methyl-CpG binding domain protein 4 | 0.0187 | 0.4733 |  | 0.7538 | 0.8425 |
| MYLK | Myosin light chain kinase. smooth muscle (MLCK) | 0.0482 | 0.4670 |  | 0.6351 | 0.7238 |
| SNX20 | Sorting nexin 20 | 0.0167 | 0.4647 |  | 0.7163 | 0.8938 |
| WNT2B | Wingless-type MMTV integration site family. member 2B | 0.0318 | 0.4618 |  | 0.6539 | 0.7595 |
| MAGEF1 | Melanoma antigen family F. 1 | 0.0307 | 0.5525 |  | 0.5837 | 0.7533 |
| ARHGAP30 | Rho GTPase activating protein 30 | 0.0212 | 0.4582 |  | 0.4961 | 0.7281 |
| VWA1 | von Willebrand factor A domain-containing protein 1 Precursor | 0.0304 | 0.4581 |  | 0.5682 | 0.7079 |
| SLC5A1 | solute carrier family 5 (sodium/glucose cotransporter). member 1 | 0.0329 | 0.4574 |  | 0.8808 | 0.8980 |
| CPPED1 | calcineurin-like phosphoesterase domain containing 1 | 0.0169 | 0.4555 |  | 0.7180 | 0.8214 |
| Amica1 | adhesion molecule. interacts with CXADR antigen 1 | 0.0026 | 0.4438 |  | 0.4088 | 0.7501 |
| PFN2 | Profilin-2 (Profilin II) | 0.0088 | 0.4353 |  | 0.4000 | 0.6735 |
| FBRSL1 | Fiibrosin-like 1 | 0.0299 | 0.4348 |  | 0.6862 | 1.3077 |
| PAG8 | Pregnancy-associated glycoprotein 8 (PAG8) | 0.0346 | 0.4273 |  | 0.5526 | 0.7022 |
| GRP116 | G protein-coupled receptor 116 (GPR116) | 0.0093 | 0.4270 |  | 0.3929 | 0.6596 |
| PDXP | Pyridoxal phosphate phosphatase (PLP phosphatase) | 0.0100 | 0.4256 |  | 0.4076 | 0.6588 |
| fgf10 | fibroblast growth factor 10 | 0.0216 | 0.4170 |  | 0.6447 | 0.7466 |
| FCGR | Low affinity immunoglobulin gamma Fc region receptor II Precursor (CD32 antigen) | 0.0088 | 0.4147 |  | 0.5142 | 0.7265 |
| ANP32A | Acidic (leucine-rich) nuclear phosphoprotein 32 family. member A | 0.0149 | 0.4105 |  | 0.8727 | 0.8966 |
| TSPAN15 | Tetraspanin-15 | 0.0239 | 0.4071 |  | 0.5746 | 1.4478 |
| cdca7 | Cell division cycle associated 7 | 0.0175 | 0.4012 |  | 0.7762 | 0.8278 |
| CAT | Catalase | 0.0067 | 0.3420 |  | 0.6443 | 0.7685 |
| CD14 | CD14 molecule | 0.0198 | 0.2693 |  | 0.5665 | 0.5921 |
